# Large-Scale Phenotyping of an Accurate Genetic Mouse Model of JNCL Identifies Novel Early Pathology Outside the Central Nervous System

**DOI:** 10.1371/journal.pone.0038310

**Published:** 2012-06-06

**Authors:** John F. Staropoli, Larissa Haliw, Sunita Biswas, Lillian Garrett, Sabine M. Hölter, Lore Becker, Sergej Skosyrski, Patricia Da Silva-Buttkus, Julia Calzada-Wack, Frauke Neff, Birgit Rathkolb, Jan Rozman, Anja Schrewe, Thure Adler, Oliver Puk, Minxuan Sun, Jack Favor, Ildikó Racz, Raffi Bekeredjian, Dirk H. Busch, Jochen Graw, Martin Klingenspor, Thomas Klopstock, Eckhard Wolf, Wolfgang Wurst, Andreas Zimmer, Edith Lopez, Hayat Harati, Eric Hill, Daniela S. Krause, Jolene Guide, Ella Dragileva, Evan Gale, Vanessa C. Wheeler, Rose-Mary Boustany, Diane E. Brown, Sylvie Breton, Klaus Ruether, Valérie Gailus-Durner, Helmut Fuchs, Martin Hrabě de Angelis, Susan L. Cotman

**Affiliations:** 1 Molecular Neurogenetics Unit, Center for Human Genetic Research, Massachusetts General Hospital, Boston, Massachusetts, United States of America; 2 Department of Pathology, Massachusetts General Hospital, Boston, Massachusetts, United States of America; 3 Institute of Developmental Genetics, Helmholtz Zentrum München, Neuherberg/Munich, Germany; 4 Department of Neurology, Friedrich-Baur-Institut, Ludwig-Maximilians-Universität München, Munich, Germany; 5 German Mouse Clinic, Institute of Experimental Genetics, Helmholtz Zentrum München, Neuherberg/Munich, Germany; 6 Charité-Eye Hospital, Campus Virchow-Klinikum, Berlin, Germany; 7 Institute of Pathology, Helmholtz Zentrum München, Neuherberg/Munich, Germany; 8 Chair for Molecular Animal Breeding and Biotechnology, Gene Center, Ludwig-Maximilians-Universität München, Munich, Germany; 9 Molecular Nutritional Medicine, Else Kröner-Fresenius Center, TUM, Freising-Weihenstephan, Germany; 10 Institute of Medical Microbiology, Immunology, and Hygiene, TUM, München, Germany; 11 Institute of Human Genetics, Helmholtz Zentrum München, Neuherberg/Munich, Germany; 12 Institute of Molecular Psychiatry, University of Bonn, Bonn, Germany; 13 Department of Medicine III, Division of Cardiology, University of Heidelberg, Otto-Meyerhof-Zentrum, Heidelberg, Germany; 14 Lehrstuhl für Entwicklungsgenetik, TUM, Freising-Weihenstephan, Germany; 15 Max-Planck-Institute of Psychiatry, Munich, Germany; 16 Deutsches Zentrum für Neurodegenerative Erkrankungen e. V. Site Munich, Munich, Germany; 17 Neurogenetics Program and Division of Pediatric Neurology, Departments of Pediatrics and Biochemistry, American University of Beirut, Beirut, Lebanon; 18 Center for Systems Biology, Program in Membrane Biology/Nephrology Division, Massachusetts General Hospital, Boston, Massachusetts, United States of America; 19 Center for Comparative Medicine, Massachusetts General Hospital, Boston, Massachusetts, United States of America; 20 Augenabteilung Sankt Gertrauden Krankenhaus, Berlin, Germany; 21 Lehrstuhl für Experimentelle Genetik, TUM, Freising-Weihenstephan, Germany; Center of Ophtalmology, Germany

## Abstract

*Cln3^Δex7/8^* mice harbor the most common genetic defect causing juvenile neuronal ceroid lipofuscinosis (JNCL), an autosomal recessive disease involving seizures, visual, motor and cognitive decline, and premature death. Here, to more thoroughly investigate the manifestations of the common JNCL mutation, we performed a broad phenotyping study of *Cln3^Δex7/8^* mice. Homozygous *Cln3^Δex7/8^* mice, congenic on a C57BL/6N background, displayed subtle deficits in sensory and motor tasks at 10–14 weeks of age. Homozygous *Cln3^Δex7/8^* mice also displayed electroretinographic changes reflecting cone function deficits past 5 months of age and a progressive decline of retinal post-receptoral function. Metabolic analysis revealed increases in rectal body temperature and minimum oxygen consumption in 12–13 week old homozygous *Cln3^Δex7/8^*mice, which were also seen to a lesser extent in heterozygous *Cln3^Δex7/8^* mice. Heart weight was slightly increased at 20 weeks of age, but no significant differences were observed in cardiac function in young adults. In a comprehensive blood analysis at 15–16 weeks of age, serum ferritin concentrations, mean corpuscular volume of red blood cells (MCV), and reticulocyte counts were reproducibly increased in homozygous *Cln3* ***^Δ^*** ^*ex7/8*^ mice, and male homozygotes had a relative T-cell deficiency, suggesting alterations in hematopoiesis. Finally, consistent with findings in JNCL patients, vacuolated peripheral blood lymphocytes were observed in homozygous *Cln3* ***^Δ^*** ^*ex7/8*^ neonates, and to a greater extent in older animals. Early onset, severe vacuolation in clear cells of the epididymis of male homozygous *Cln3* ***^Δ^*** ^*ex7/8*^ mice was also observed. These data highlight additional organ systems in which to study CLN3 function, and early phenotypes have been established in homozygous *Cln3* ***^Δ^*** ^*ex7/8*^ mice that merit further study for JNCL biomarker development.

## Introduction

The neuronal ceroid lipofuscinoses (NCLs, also known as Batten disease) are a genetically heterogeneous group of rare, inherited lysosomal disorders that are typified primarily by CNS features, including progressive vision loss, dementia, seizures, loss of motor coordination, gliosis and neuronal atrophy, culminating in premature death [1]. The hallmark pathology, ceroid lipofuscin lysosomal storage material, is observed in most cells, suggesting that there may be unrecognized functional deficits outside of the CNS in NCL patients. Indeed, a number of case reports have documented cardiac defects in juvenile NCL (JNCL) patients and several recent larger studies further support an association between JNCL and cardiac dysfunction [2], [3], [4]. Immune system abnormalities have also been documented in JNCL patients [5], [6].

Mutations in *CLN3*, localized to chromosome 16p11.2, are responsible for JNCL, which presents clinically between 4 and 10 years of age [7]. Most JNCL patients are homozygous for a 1.02-kb genomic deletion, which evidence suggests leads to aberrant mRNA transcripts encoding truncated and internally deleted mutant CLN3 protein variants [7], [8]. More than 50 additional *CLN3* mutations have now been documented, including nonsense, missense, frameshift, and splice site mutations (http://www.ucl.ac.uk/ncl/cln3.shtml), which are most often compound heterozygous with the common 1.02-kb deletion, but are also occasionally seen in a heterozygous state with other rare mutations [9], [10]. Though CLN3 function itself is not yet fully delineated, numerous studies strongly suggest that CLN3, a primarily endosomal-lysosomal protein in mammalian cells, plays a major role in post-Golgi, endocytic, autophagic and lysosomal trafficking [for reviews, see [11], [12]], possibly via regulating membrane lipid content [13], vesicular pH [14], and/or via regulation of palmitoylated signaling proteins which may in turn regulate trafficking [15], [16].

In addition to the lower eukaryotic CLN3-deficiency yeast models that have significantly expanded our current understanding of CLN3 function [14], [16], [17], [18], four mouse models of JNCL have been established and characterized to varying degrees [reviewed in [19]]. Two different *Cln3* ‘knock-out’ models were created by replacing different portions of the murine *Cln3* gene with a neomycin resistance cassette [20], [21]. To facilitate simultaneous study of *in vivo Cln3* gene expression and deficiency phenotypes, a *Cln3* reporter mouse model was also established in which exons 1–8 were replaced by a *lacZ* reporter gene [22]. Finally, we previously utilized homologous recombination and Cre-*lox* P-mediated technology to create a ‘knock-in’ mouse in which the common ∼1-kb deletion was introduced into the endogenous murine *Cln3* gene [8].

Consistent with the predicted loss of CLN3 normal function as the root cause of this recessively inherited disease, all of the established mouse models display recessive features of JNCL including accumulation of ceroid lipofuscin, brain gliosis, neurological dysfunction and neurodegeneration [8], [20], [21], [22], [23], [24], [25]. Whether there are differences across the models in the specific behavioral abnormalities or in timing of disease onset and progression is unclear, as specific comparisons across the different *Cln3* mouse studies are confounded by differences in genetic background, environment, and methodology. Nevertheless, the *Cln3* ***^Δ^*** ^*ex7/8*^ knock-in mouse represents the only genetically accurate JNCL mouse model, and therefore may be most predictive of the earliest molecular and cellular consequences of *CLN3* mutation in JNCL [8].

Homozygous *Cln3* ***^Δ^*** ^*ex7/8*^ mice, first characterized on an outbred CD1 background, mixed with 129 Sv/Ev, display the JNCL hallmark lysosomal storage pathology before birth, in subsets of cells in both CNS and non-CNS tissues [8]. Homozygous mutant mice otherwise appear normal at birth, but at later ages exhibit neurological abnormalities, detectable as an increased tendency to clasp the hind limbs when suspended by the tail and as an altered gait, measured in a quantitative gait analysis at 10–12 months of age, compared to wild-type and heterozygous littermate mice [8]. Aged homozygous *Cln3* ***^Δ^*** ^*ex7/8*^ mice also die prematurely, although the proximal cause of death is not known [8]. Obvious seizures have not been observed in these mice. However, a thorough analysis of brain electrical activity by electroencephalography has not yet been performed. Intriguingly, homozygous *Cln3* ***^Δ^*** ^*ex7/8*^ mice were also shown to have a delay in axon pruning at the neuromuscular junction [26], and behavioral phenotypes consistent with a neurodevelopmental delay have been reported [27]. Neuropathologic studies have revealed indications of oxidative stress and lowered NMDA and M1 muscarinic acetylcholine receptor binding in the hippocampal and cortical regions of brain sections from 5-month-old homozygous *Cln3* ***^Δ^*** ^*ex7/8*^ mice [24]. Moreover, brains from 12-month-old mice on the outbred CD1 background displayed widespread gliosis, neuronal loss in the thalamocortical brain nuclei [25] and in the retina [8]. Together, these data establish JNCL neurodegenerative disease hallmarks and a functional decline that is ongoing in aging homozygous *Cln3* ***^Δ^*** ^*ex7/8*^ mice, confirming the usefulness of this accurate genetic model for JNCL research.

A more thorough knowledge of the early stages of the disease process in *Cln3* ***^Δ^*** ^*ex7/8*^ mice on a genetically defined background will be invaluable to future disease modifier studies and could lead to new biomarker tools. Therefore, here, we have analyzed young adult *Cln3* ***^Δ^*** ^*ex7/8*^ mice, inbred on a C57BL/6N background, for their overall health and organ systems functions, employing a phenotyping workflow previously established by the German Mouse Clinic [www.mouseclinic.de] [28], [29], [30]. The results described in this report, while augmenting existing knowledge of the CNS manifestations of the common JNCL mutation, also strongly implicate important roles for CLN3 outside of the CNS, laying the groundwork for new biomarker development.

## Results

### Neurological and Behavioral Abnormalities in Young Adult Homozygous Cln3***^Δ^*** ^ex7/8^ Mice

As summarized in Table 1, young adult wild-type, heterozygous and homozygous *Cln3* ***^Δ^*** ^*ex7/8*^ mice congenic on the C57BL/6N background were subjected to a battery of behavioral and neurological tests to broadly assay for abnormalities that may be associated with the early stages of the JNCL-like disease resulting from the common *Cln3* ∼1-kb deletion mutation (see Methods).

**Table 1:** Summary of neurological and behavioral testing of *Cln3* **^Δ^** ^*ex7/8*^ mice.

Test	Age	Results
**Open Field**	10 weeks-males	Genotype effect on time spent in center (*Cln3^Δex7/8^* heterozygotes only); Trend of reduced habituation (*Cln3^Δex7/8^* homozygotes)
	11 weeks-females	Genotype effect on time spent in center (*Cln3^Δex7/8^* heterozygotes only); Trend of reduced habituation (*Cln3^Δex7/8^* homozygotes)
**Modified SHIRPA**	10 weeks-males	no genotypic difference
	11 weeks-females	Genotype effect on touch escape behavior (*Cln3^Δex7/8^* heterozygotes and homozygotes)
**Grip Strength**	10 weeks-males	no genotypic difference
	11 weeks-females	no genotypic difference
**Rotarod**	11 weeks-males	no genotypic difference
	12 weeks-females	no genotypic difference
**Pole Climbing**	11 weeks-males	no genotypic difference
	12 weeks-females	Genotype effect on total time to descend pole (*Cln3^Δex7/8^* homozygotes)
**PPI and Acoustic Startle**	12 weeks-males	Genotype effect on percentage PPI (*Cln3^Δex7/8^* homozygotes compared to *Cln3^Δex7/8^* heterozygotes)
	13 weeks-females	Genotype effect on acoustic startle (*Cln3^Δex7/8^* homozygotes)
**Nociception-Hot Plate**	13 weeks-males	no genotypic difference
	14 weeks-females	Genotype effect on time to first sign of pain (*Cln3^Δex7/8^* homozygotes)

A summary of genotypic differences observed in the neurological and behavioral screens is shown, with male and female results shown separately. Ages at which the indicated tests were performed are also shown.

Tests of the motor function and exploratory behavior of homozygous *Cln3^Δex7/8^* mice, compared to wild-type and heterozygous *Cln3^Δex7/8^* littermate mice, were first assessed at 10–11 weeks of age by open field analysis, modified SHIRPA analysis [30], [31] and grip strength tests. Subsequently, mice were tested on an accelerating rotarod (11–12 weeks of age) and in pole climbing (12–13 weeks of age) tests (Table 1).

In the open field analysis, only minor genotypic differences were observed. Male and female homozygous *Cln3* **^Δ^** ^*ex7/8*^ mice did not perform differently from wild-type littermates in distance traveled (total or center), rearing frequency, or time spent in the center, though we noted a trend of reduced habituation over the 20-minute trial, compared to wild-type or heterozygous *Cln3* **^Δ^** ^*ex7/8*^ littermates (ANOVA, p = 0.059 for females and p = 0.096 for males; Fig. S1). Interestingly, male and female heterozygous *Cln3* **^Δ^** ^*ex7/8*^ mice also tended to spend more time in the center of the open field chamber, compared to wild-type littermates (Fig. S1). It is noteworthy that our results are not in agreement with the findings reported by Osorio et al [27] in which 8-week-old homozygous *Cln3* **^Δ^** ^*ex7/8*^ mice on a C57BL/6J background were found to have reduced exploratory activity (reduced rearing frequency and vertical locomotion) and to spend less time in the center of the chamber in an open field assay.

No genotypic differences were observed in grip strength (Fig. S2), and homozygous *Cln3* **^Δ^** ^*ex7/8*^ mice overall behaved normally in the modified SHIRPA analysis (data not shown), though a minor difference was observed in the touch escape behavior of female heterozygous and homozygous *Cln3* **^Δ^** ^*ex7/8*^ mice, who displayed a decreased tendency to flee prior to touch (10% and 20% of females fled prior to touch, respectively) compared to wild-type female littermates (40% fled prior to touch; Chi-square test, p<0.05).

**Figure 1:**
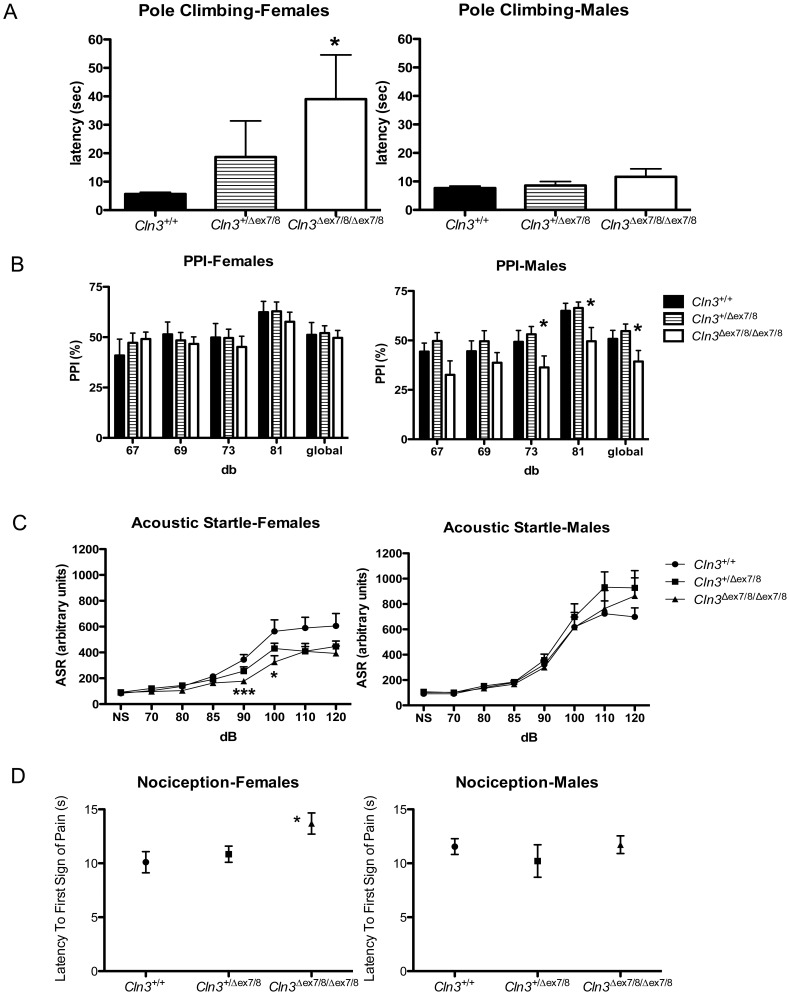
Subtle genotypic differences in performance of young adult *Cln3* ^Δ^ ^***ex7/8***^ **mice in sensory and motor neurological assays.** Shown are results of behavioural analyses in a vertical pole-climbing test (A), prepulse inhibition to the acoustic startle response (PPI) (B), acoustic startle response (C), and thermal nociception (D) for female (left) and male (right) littermate control (*Cln3^+/+^*), heterozygous (*Cln3^+/^* ^**Δ***ex7/8*^) and homozygous (*Cln3* **^Δ^** ^*ex7/8/***Δ***ex7/8*^) mice (n = 9–10 mice per group). Data are presented as mean ± standard error of the mean (SEM). (A) Homozygous *Cln3* **^Δ^** ^*ex7/8*^ female mice had an increased latency to descend the pole, compared to female wild-type or heterozygous littermates. In a Kruskal-Wallis test, the genotype effect was p<0.01 (*) for females, with or without heterozygous *Cln3* **^Δ^** ^*ex7/8*^ mice included in the analysis. (B) Mean %PPI to an acoustic startle, with four prepulse intensities (67, 69, 73, 81 decibels [db]), or with all prepulse intensities averaged (‘global’) are shown. *, ANOVA, p<0.05. (C) The mean ± SEM of the acoustic startle response to 70–120 db sounds is shown for littermate control (*Cln3^+/+^*, circles), heterozygous (*Cln3^+/^* ^**Δ***ex7/8*^, squares) and homozygous (*Cln3* **^Δ^** ^*ex7/8/***Δ***ex7/8*^, triangles) *Cln3* **^Δ^** ^*ex7/8*^ mice. NS = no startle sound. For females, ANOVA, genotype effect was F_(7,11)_ = 4.63, p<0.05, and post-hoc tests revealed that this was significant at 90 and 100 db (*p<0.05, ***P<0.001). No statistically significant differences were detected in the acoustic startle response of males. (D) The mean ± SEM latency to the first sign of pain (seconds = s) in a hot plate assay is shown. *, ANOVA genotype effect p<0.05.

Again in contrast to the Osorio et al study [27], in which 8-week homozygous *Cln3* **^Δ^** ^*ex7/8*^ mice on a C57BL/6J background were reported to perform more poorly than wild-type mice on an accelerating rotarod, we found no genotypic differences in accelerating rotarod performance in 10- to 11-week-old *Cln3* **^Δ^** ^*ex7/8*^ mice on the C57BL/6N background (Fig. S3). However, in a second test of motor coordination, the pole-climbing test, where mice were placed at the top of a round, metal bar, head upwards, and time-to-turn and time-to-descend the bar were recorded, especially female homozygous *Cln3* **^Δ^** ^*ex7/8*^ mice performed significantly worse than wild-type or heterozygous female littermate mice (Fig. 1A). Notably, the methodology and apparatus used in the open field and rotarod assays in this study and in the Osorio et al. study differed [see Methods and [27]].

We further tested *Cln3* **^Δ^** ^*ex7/8*^ mice in several sensorimotor tasks, including in acoustic startle and its pre-pulse inhibition (PPI) (12–13 weeks of age) and nociception hot-plate (13–14 weeks of age) assays (Table 1). Homozygous *Cln3* **^Δ^** ^*ex7/8*^ male mice displayed reduced PPI (p<0.05) compared to heterozygous littermates at 73 decibel (dB), 81 dB, and when all four pre-pulse intensities were averaged (global), which was not a consequence of hearing loss since the acoustic startle reactivity of homozygous *Cln3* **^Δ^** ^*ex7/8*^ male mice did not differ from heterozygous or wild-type littermates (Fig. 1B, C). Female homozygous *Cln3* **^Δ^** ^*ex7/8*^ mice did not show the same reduction in PPI, but did display reduced startle reactivity compared to wild-type littermates (ANOVA, genotype effect: F_(7,11)_ = 4.63, p<0.05) (Fig. 1C). Finally, in the nociception hot-plate assay, we noted a slight increase in the latency to the first sign of pain in response to heat stimuli in female homozygous *Cln3* **^Δ^** ^*ex7/8*^mice (p<0.05), though no changes in this reaction were detected in the male homozygous *Cln3* **^Δ^** ^*ex7/8*^ mice (Fig. 1D). Taken together, these data suggest that homozygous *Cln3* **^Δ^** ^*ex7/8*^ mice on the C57BL/6N background harbor early, subtle defects in sensory and motor functions.

To determine whether neurodegenerative changes were also present in the young adult *Cln3* **^Δ^** ^*ex7/8*^ mice, brains were isolated from 20-week-old mice for subsequent morphological and immunohistochemical assessment, probing the extent of neuronal cell death, lysosomal storage, and gliosis. Gross morphological assessment of brains indicated no obvious neuronal cell loss in homozygous *Cln3* **^Δ^** ^*ex7/8*^ mice, as brain sizes were not different (Figs. S4) and TUNEL staining, which marks apoptotic nuclei, was negative (data not shown). These results were consistent with our previous studies of homozygous *Cln3* **^Δ^** ^*ex7/8*^ mice on the mixed 129 Sv/Ev/CD1 background, in which only at later ages (12 months) did we see any significant neuronal cell loss accompanied by minor decreases in total brain weight [8], [24], [25].

The pathological storage material in 20-week-old homozygous *Cln3* **^Δ^** ^*ex7/8*^ mice on the C57BL/6N background was analyzed by immunostaining for the mitochondrial ATP synthase subunit c protein, which is the main proteolipid found in the lysosomal deposits [32]. Subunit c storage was evident in selected neuronal populations across multiple brain regions including the hippocampus, thalamus, cortex, amygdala, and cerebellum (Fig. S5 and data not shown), in a pattern that was consistent with our previously published data on brain pathology in homozygous *Cln3* **^Δ^** ^*ex7/8*^ mice on the CD1 background [8].

We also surveyed relative astrocytosis in the brains of the 20-week-old *Cln3* **^Δ^** ^*ex7/8*^ mice on the C57BL/6N background, by immunostaining with antibodies recognizing glial fibrillary acidic protein (GFAP) and S100, widely used astrocyte markers. No obvious genotypic differences were observed in the GFAP immunostained brain sections from 20-week old homozygous *Cln3* **^Δ^** ^*ex7/8*^ and wild-type littermate mice (data not shown), while subtle differences in the S100 staining results were observed (Fig. S6). S100 immunostain was broadly darker in the homozygous *Cln3* **^Δ^** ^*ex7/8*^ mouse brain sections, particularly in the neuropil, compared to the staining observed in the brain sections from wild-type littermate mice (Fig. S6). Thus, these data suggest that behavioral abnormalities suggestive of sensory and motor defects in young adult homozygous *Cln3* **^Δ^** ^*ex7/8*^ mice are not a result of neuronal cell loss, but rather that they signify an early functional decline in brain circuitry that is yet to be fully elucidated.

### Late Onset Retinal Degeneration in Homozygous Cln3**^Δ^** ^ex7/8^ Mice

Funduscopy, slit lamp microscopy and laser interference biometry examinations were performed on homozygous *Cln3* **^Δ^** ^*ex7/8*^ mice, and heterozygous and wild-type littermates, at 15 weeks of age. No significant genotypic differences were detected in the appearance of the fundus or the anterior and posterior segments of the retina, and the axial eye length was not different (Table S1).

**Figure 2:**
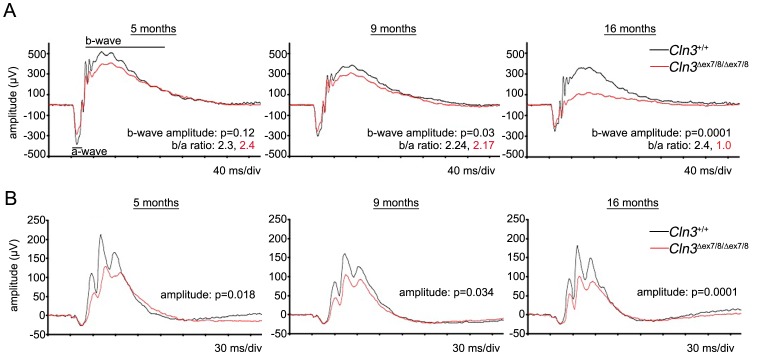
Electroretinography of 16-month-old *Cln3* ^Δ^ ^***ex7/8***^ **mice.** (A) Scotopic ERG traces are shown for 5-, 9-, and 16-month old wild-type (*Cln3^+/+^*, black trace, n = 7) and homozygous *Cln3* **^Δ^** ^*ex7/8*^ (*Cln3* **^Δ^** ^*ex7/8/***Δ***ex7/8*^, red trace, n = 8) mice. The relative amplitudes of the a-wave do not dramatically differ between the wild-type and homozygous *Cln3* **^Δ^** ^*ex7/8*^ mice. However, the b-wave is drastically reduced in aged homozygous *Cln3* **^Δ^** ^*ex7/8*^ mice, compared to wild-type littermates. Thus, homozygous *Cln3* **^Δ^** ^*ex7/8*^ mice exhibit an electronegative ERG at 16-months of age (b/a ratio = 1, versus b/a ratio = 2.4 in wild-type mice). (B) Photopic ERG traces, reflecting cone response, are shown for 5-, 9-, and 16-month-old wild-type (*Cln3^+/+^*, black trace, n = 7) and homozygous *Cln3* **^Δ^** ^*ex7/8*^ (*Cln3* **^Δ^** ^*ex7/8/***Δ***ex7/8*^, red trace, n = 8) mice. There was a significant genotypic difference in the relative mean amplitudes already at 5 months of age.

Study of retinal function in *Cln3* **^Δ^** ^*ex7/8*^ mice has not previously been reported for any genetic background, but our previous morphological analysis of the retina from aged homozygous *Cln3* **^Δ^** ^*ex7/8*^ mice outbred on the CD1 background indicated a low level cell loss within the retina in hypopigmented mice, without dramatic thinning of the retina [8]. Subsequent studies of the CD1 background mice indicated additional retinal degeneration genetic loci, independent of the *Cln3* locus, leaving the precise details of retinal degeneration as a result of the *Cln3* **^Δ^** ^*ex7/8*^ mutation in question (Ruether, unpublished data). Therefore, we sought to further evaluate the vision of aging *Cln3^?ex7/8^* mice congenic on the C57BL/6N background by electroretinography (ERG) at 5, 9, and 16 months of age. The scotopic (dark-adapted) ERG reflecting rod function and, at higher stimulus strengths, mixed rod-cone function, shows a progressive decline of the b-wave amplitude of homozygous *Cln3^Δex7/8^* mice, reaching statistical significance at an age of 9 months compared to wild-type littermates (Fig. 2A). At an age of 16 months the difference was profound. However, there was virtually no difference between a-wave amplitude at any of the ages. At 16 months of age, the b/a ratio for homozygous *Cln3* **^Δ^** ^*ex7/8*^ mice was 1.0, indicating the a- and b-wave ERG components had the same amplitudes. In normal mice, the b/a wave ratio is typically greater than 1.6. By photopic (light-adapted) ERG, which primarily reflects cone function, amplitudes were already significantly reduced in homozygous *Cln3* **^Δ^** ^*ex7/8*^ mice by the age of 5 months, compared to wild-type littermates, and further reduction in the amplitude measured in homozygous *Cln3* **^Δ^** ^*ex7/8*^ mice was observed at 16 months of age (Fig. 2B). The a-wave of the ERG originates in the photoreceptor layer, while the b-wave emanates from lower order retinal cells, postsynaptic to the photoreceptors [33]. Therefore, the selective loss of the b-wave in homozygous *Cln3* **^Δ^** ^*ex7/8*^ mice on the C57BL/6N background, indicates that there is primarily a loss of function in the postsynaptic retinal neurons.

### Abnormal Metabolism in Young Adult Cln3**^Δ^** ^ex7/8^ Mice

**Figure 3:**
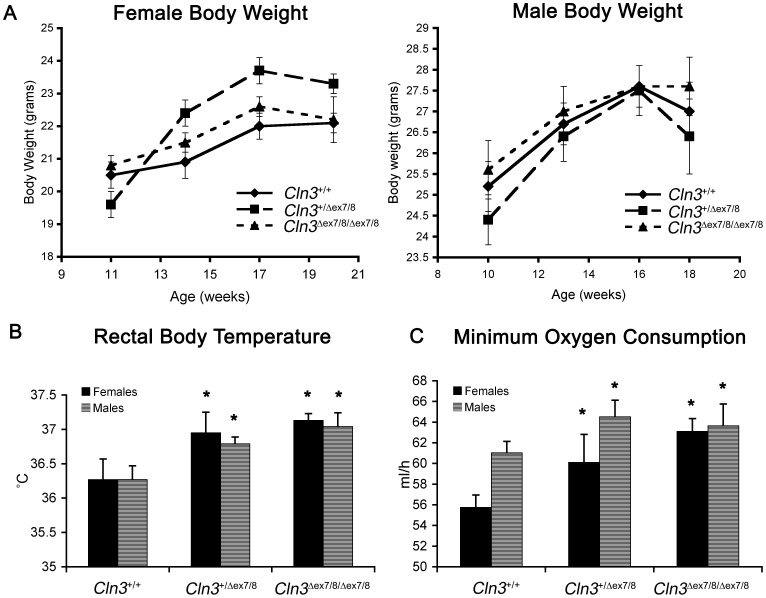
Metabolic abnormalities in *Cln3* ^Δ^ ^***ex7/8***^ **mice.** (A) Graphs depicting female (left) and male (right) mean body weight data from wild-type (diamonds), heterozygous (squares), and homozygous (triangles) *Cln3* **^Δ^** ^*ex7/8*^ mice at ages between 11 and 20-weeks are shown (n = 5–10 mice per genotype/sex/age). No significant genotypic differences were observed. Error bars represent SEM. (B) Mean ± SEM rectal body temperatures are shown for male (black bars) and female (gray bars) wild-type (*Cln3^+/+^*), heterozygous (*Cln3^+/^* ^**Δ***ex7/8*^) and homozygous (*Cln3* **^Δ^** ^*ex7/8/***Δ***ex7/8*^) littermate mice are shown. Rectal body temperatures, which were measured at rest, were slightly elevated in male and female, heterozygous and homozygous *Cln3* **^Δ^** ^*ex7/8*^ mice, compared to wild-type mice. *, p<0.001 (heterozygous versus wild-type, homozygous versus wild-type). (C) Mean ± SEM values for minimum oxygen consumption (ml/hr) are shown for male (black bars) and female (gray bars) wild-type (*Cln3^+/+^*), heterozygous (*Cln3^+/^* ^**Δ***ex7/8*^) and homozygous (*Cln3* **^Δ^** ^*ex7/8/***Δ***ex7/8*^) littermate mice are shown. Minimum oxygen consumption was elevated in male and female heterozygous and homozygous *Cln3^Δex7/8^* mice, compared to wild-type mice. 5–10 mice per group (genotype/sex) were analyzed. *, p<0.001 (heterozygous versus wild-type, homozygous versus wild-type).

To monitor overall health in young adult *Cln3* **^Δ^** ^*ex7/8*^ mice on the C57BL/6N background, body weight between 10 and 20 weeks of age was monitored for wild-type, heterozygous, and homozygous *Cln3* **^Δ^** ^*ex7/8*^ mice maintained on a normal diet (5% crude fat); genotype had no significant effect on body weight (Fig. 3A). To further assess overall energy metabolism, 13-week-old male and 14-week-old female mice, were monitored by indirect calorimetry for a 21-hour period, during a 12-hour light-dark cycle (see Methods). *Cln3* **^Δ^** ^*ex7/8*^ mutant mice did not display differences in activity, food consumption, or mean respiratory exchange ratio (Table S2). However, rectal body temperature, measured late-morning at the end of the testing period when mice were at rest, and minimum oxygen consumption were significantly elevated in heterozygous and homozygous *Cln3* **^Δ^** ^*ex7/8*^ mice (Fig. 3B, C).

**Figure 4:**
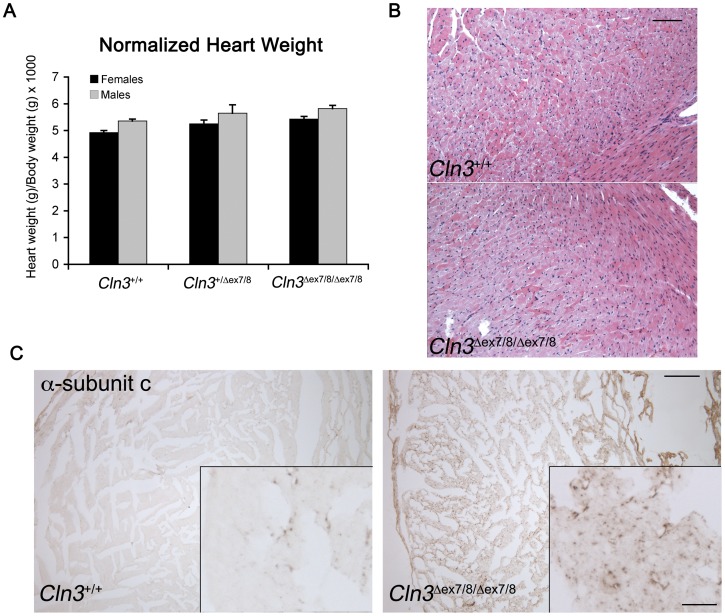
Heart analysis of *Cln3* ^Δ^ ^***ex7/8***^ **mice.** (A) The bar graph depicts normalized heart weights for wild-type (*Cln3^+/+^*), heterozygous (*Cln3^+/^* ^**Δ***ex7/8*^), and homozygous (*Cln3* **^Δ^** ^*ex7/8/***Δ***ex7/8*^) littermate 19–20 week old mice. Normalized heart weights represent a ratio of heart weight (mg = milligrams)/body weight (g = grams). Normalized heart weights were slightly increased in heterozygous *Cln3* **^Δ^** ^*ex7/8*^ mice, and more so in homozygous *Cln3* **^Δ^** ^*ex7/8*^ mice, compared to wild-type littermates. ANOVA analysis suggested a significant genotype effect (p<0.05). (B) Representative micrographs of hematoxylin and eosin (H&E) stained heart sections from wild-type (*Cln3^+/+^*, n = 8) and homozygous (*Cln3* **^Δ^** ^*ex7/8/***Δ***ex7/8*^, n = 10) littermate 19–20 week old mice are shown, which do not obviously differ from one another in their morphology. Scale bar = 100 µm. (C) Representative micrographs are shown of α-subunit c immunostained heart sections from 19-week old *Cln3^+/+^* and *Cln3* **^Δ^** ^*ex7/8/***Δ***ex7/8*^ littermate mice. Note the abundance of subunit c-immunopositive deposits in the *Cln3^?ex7/8/^* ^**Δ***ex7/8*^ section. Only sparse punctate subunit c immunostaining is present in the *Cln3^+/+^* section. Scale bar = 200 µm. Inset scale bar = 25 µm.

Increasing evidence indicates abnormal cardiovascular health in JNCL patients [3], [4]. To assess the cardiovascular status of *Cln3* **^Δ^** ^*ex7/8*^ mice, blood pressure (12–13 weeks of age), pulse rate (16–17 weeks of age), echocardiography parameters (16–17 weeks of age), and serum N-terminal pro atrial natriuretic peptide (Nt-proANP) levels (at 18–19 weeks of age) were measured and analyzed. None of these parameters were significantly altered in heterozygous or homozygous *Cln3* **^Δ^** ^*ex7/8*^ mutant mice at these ages, compared to wild-type littermate mice (see Figs. S7, S8, Table S3). However, normalized heart weight, measured at 20 weeks of age, was slightly increased in heterozygous and homozygous *Cln3* **^Δ^** ^*ex7/8*^ mice, compared to wild-type littermate mice (ANOVA, p<0.05; Fig. 4A). Despite this difference, in further histological assessment of heart from 19-week-old mice, we did not detect any obvious signs of pathological cardiac hypertrophy (Fig. 4B). Not surprisingly, subunit c-positive lysosomal storage material was evident in homozygous *Cln3* **^Δ^** ^*ex7/8*^ mice (Fig. 4C), while no storage material was observed in *Cln3* **^Δ^** ^*ex7/8*^ heterozygotes (data not shown). We also immunostained heart sections with an antibody recognizing nuclear factor of activated T-cells (NFAT), which is a central regulator of the signaling pathways mediating cardiac hypertrophy and enters the nucleus upon activation of hypertrophic signaling pathways [34]. No significant differences in nuclear NFAT levels were detected in heart sections from homozygous *Cln3* **^Δ^** ^*ex7/8*^ mice, compared to those from wild-type littermate mice (data not shown). Therefore, these results suggest that the common JNCL mutation in the mouse leads to an increased resting metabolism, without significant differences in overall cardiovascular function in young adults.

### Abnormalities in Blood Chemistry and Hematological Parameters in Young Adult Cln3*^?^* ^ex7/8^ Mice

Leukocyte populations in peripheral blood isolated from 15- to 16-week-old *Cln3* **^Δ^** ^*ex7/8*^ mice and wild-type littermates were analyzed by flow cytometry, and plasma levels of immunoglobulins were measured. No significant differences in plasma immunoglobulins were detected across the samples analyzed (data not shown). We did not specifically assay serum from *Cln3* **^Δ^** ^*ex7/8*^ mice for the absence or presence of autoantibodies to GAD65 and alpha-fetoprotein, which have been reported in serum from *Cln3* knock-out mice and JNCL patients [5], [6].

By flow cytometry, we observed a significantly lower frequency of T cells in male heterozygous and homozygous *Cln3* **^Δ^** ^*ex7/8*^ mice, compared to controls, but no genotypic differences were observed in the overall T cell frequency among female mice (Table 2). The relative proportions of the CD4+ and CD8+ T cell populations were also altered in male heterozygous and homozygous *Cln3* **^Δ^** ^*ex7/8*^ mice; the ratio of CD4+/CD8+ T cells was significantly reduced in male homozygous *Cln3* **^Δ^** ^*ex7/8*^ mice and tended to be lower in male heterozygous *Cln3* **^Δ^** ^*ex7/8*^ mice (Table 2). Moreover, we observed a higher proportion of Ly6C-expressing cells within the CD4+ and CD8+ T cell clusters in samples from male heterozygous and homozygous *Cln3* **^Δ^** ^*ex7/8*^ mice (Table 2). Ly6C is a surface molecule expressed especially on central memory T cells [35]. Notably, the CD4+/CD8+ ratio was also significantly reduced in the female homozygous *Cln3* **^Δ^** ^*ex7/8*^ mice, though no other genotypic differences among the other leukocyte populations were observed among the female mice (Table 2).

**Table 2:** T cell frequencies in peripheral blood from *Cln3* **^Δ^** ^*ex7/8*^ mice.

	Genotype	% T cells (CD45+)	CD4+/CD8+	% Ly6C+ cells among CD8+ population	% Ly6C+ cells among CD4+ population
**Females**	*Cln3^+/+^* (n = 10)	24.6	1.1	36.1	44.5
	*Cln3^+/Δex7/8^* (n = 10)	24.8 (p = 0.902)	1.07 (p = 0.572)	37.5 (p = 0.409)	47.7 (p = 0.122)
	*Cln3^Δex7/8/Δex7/8^* (n = 9)	24.7 (p = 0.961)	**0.987 (p = 0.013)**	37.1 (p = 0.548)	48.4 (p = 0.097)
**Males**	*Cln3^+/+^* (n = 10)	17.2	1.2	37.1	42.8
	*Cln3^+/Δex7/8^* (n = 9)	**14.1 (p = 0.026)**	1.06 (p = 0.071)	**43.3 (p = 0.025)**	**45.9 (p = 0.029)**
	*Cln3^Δex7/8/Δex7/8^* (n = 10)	**14.4 (p = 0.027)**	**1.03 (p = 0.002)**	**43.4 (p = 0.002)**	**47.5 (p = 0.002)**

The frequencies of T-cells [% T cells (CD45+)], the ratios of CD4+/CD8+ T cells, and the percentage of Ly6c+ cells among the CD8+ and CD4+ T cell populations, determined by flow cytometry, are shown for female and male wild-type (*Cln3^+/+^*), heterozygous (*Cln3^+/^* ^**Δ***ex7/8*^), and homozygous (*Cln3^Δex7/8/^* ^**Δ***ex7/8*^) littermate mice. p values, determined in a two-tailed, unpaired Student’s t-test of the heterozygous *Cln3* **^Δ^** ^*ex7/8*^ values versus wild-type (*Cln3^+/+^*) values, or homozygous *Cln3* **^Δ^** ^*ex7/8*^ values versus wild-type (*Cln3^+/+^*) values, are shown. Bold typeface highlights parameters that were significantly different versus wild-type controls. Samples from 9–10 mice per group (genotype/sex) were analyzed, as indicated.

Sex-dependent differences in the frequencies of leukocyte subsets are known in inbred strains of mice and are considered to be biologically relevant, as they reflect sex differences in the susceptibility to autoimmunity or infection [36]. Under baseline conditions, in many inbred strains, the frequency of T cells in peripheral blood is higher in females than in male mice [37]. This was also the case in the analyzed cohort of *Cln3* **^ΔΔ^** ^*ex7/8*^ mice and littermate controls.

**Figure 5:**
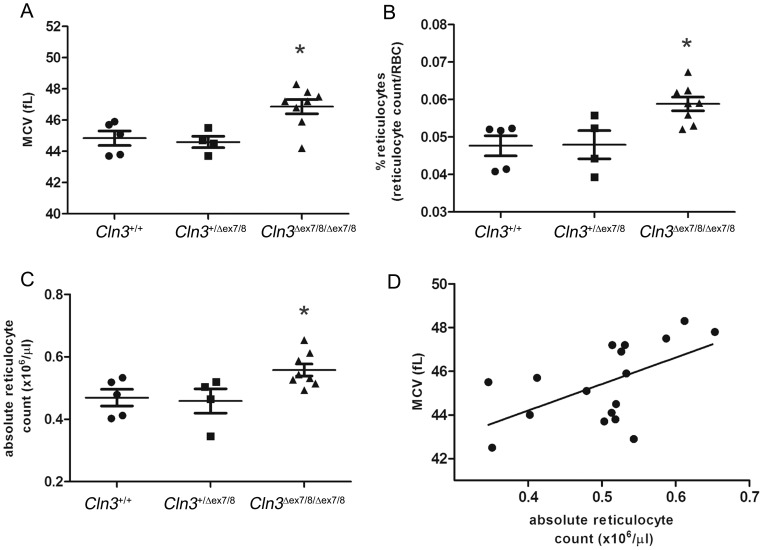
Abnormal hematology in peripheral blood from homozygous *Cln3* ^Δ^ ^***ex7/8***^ **mice.** (A) Mean corpuscular volume (MCV, fL) of peripheral red blood cells from ∼12-week-old mice was measured on an automated analyzer. *, p<0.05, WT and heterozygous mutant mice vs. homozygous mutant mice, unpaired, two-tailed *t* test. Data shown as mean ± SEM. Percentage of reticulocytes (B) and absolute reticulocyte counts (C) on the specimens analyzed in (A) were determined manually by new methylene blue staining. *, p<0.05, WT and heterozygous mutant mice vs. homozygous mutant mice, unpaired, two-tailed *t* test. Data shown as mean ± SEM. (D) Linear regression analysis of data from (A) and (C). *r^2^* = 0.32, p = 0.02. Datapoints represent individual mice.

To survey *Cln3* **^Δ^** ^*ex7/8*^ mice in standard clinical chemistry and hematological parameters, we collected blood samples from 12- to 19-week-old wild-type, heterozygous and homozygous littermate *Cln3* **^Δ^** ^*ex7/8*^ mice. Samples were analyzed for 21 different analytes including plasma electrolytes, liver enzymes, ferritin and transferrin, and for basic hematological and immunological parameters. No differences in plasma electrolytes or liver enzyme activities were observed. In contrast, serum ferritin concentrations were consistently elevated in homozygous *Cln3* **^Δ^** ^*ex7/8*^ mice compared to wild-type and heterozygous littermates (Tables 3 and S4). We also observed a consistently increased mean corpuscular volume (MCV) in the complete blood count (CBC) analysis from homozygous *Cln3* **^Δ^** ^*ex7/8*^ mice, as compared to wild-type and heterozygous *Cln3* **^Δ^** ^*ex7/8*^ littermate mice (Tables 3 and S4, Fig. 5A).

**Table 3:** Blood analysis of *Cln3* **^Δ^** ^*ex7/8*^ mice.

Analyte/Parameter	Males			Females		
	*Cln3^+/+^*	*Cln3^+/Δex7/8^*	*Cln3^Δex7/8/Δex7/8^*	*Cln3^+/+^*	*Cln3^+/Δex7/8^*	*Cln3^Δex7/8/Δex7/8^*
Ferritin (ng/ml)	31.2±2.1	24.6±1.8	35.6±1.9**	24.2±2.8	27.4±1.8	33.1±6.4**
	21.2±1.3	21.5±1.5	29.6±1.95**	20.9±2.0	44.3±17.5	30.7±1.8**
RBC (10^6^/µl)	11.34±0.15	10.98±0.19	11.27±0.12	10.48±0.12	10.86±0.08	10.58±0.12
	10.45±0.26	10.09±0.59	10.57±0.12	10.4±0.31	9.82±0.3	10.06±0.4
	*10.58±0.87*	*10.63±1.04*	*10.77±0.84*	*10.12±0.79*	*10.32±1.08*	*10.47±0.91*
MCV (fl)	49.3±0.3	50.1±0.26	52±0.3**	49.9±0.2	51.6±0.67	52.8±0.46**
	50.9±0.35	50.5±0.46	52.4±0.65**	51.2±0.75	51.9±0.54	54±0.63**
	*45.3±0.79*	*44.6±0.82*	46.9±0.64*	*44.8±0.84*	*44.7±0.89*	47.1±0.91*
Retic. Count (10^6^/µl)	*0.47±0.08*	*0.45±0.12*	0.56±0.1*	*0.48±0.09*	*0.41±0.18*	0.57±0.11*
RDW (% of MCV)	12.9±0.07	12.7±0.1	12±0.11**	13±0.13	12.8±0.08	12.1±0.12**
	13.8±0.14	13.7±0.08	13.3±0.15**	13.8±0.17	13.6±0.13	13.2±0.15**
	*19.8±0.24*	*20.6±0.29*	*20.3±0.26*	*20.7±0.23*	*19.9±0.21*	*20.5±0.27*

The subset of clinical chemistry and hematological parameters that were found to have genotype-specific differences in the screen are shown, with significantly different values indicated in bold. RBC count, which did not differ, is also shown. Each row of values represents an independent set of measurements. Data represent mean ± SEM. *p<0.05, **p<0.01 (2-way ANOVA for each set of measurements). For the statistical analysis of ferritin levels, one mouse from the group of heterozygous (*Cln3^+/^* ^**Δ***ex7/8*^) females was excluded as an outlier. Non-italicized values were determined at the German Mouse Clinic. Italicized values were determined at Massachusetts General Hospital on a separate cohort of mice. The ∼5–6 fL offset in MCV measurements between these sites, as well as the ∼6–7% offset in RDW measurements, are likely due to differences in the automated analyzers used. RBC = red blood cell count, MCV = mean corpuscular volume, Retic. = reticulocyte, RDW = red cell distribution width. Two separate samples from 9–10 mice per group (genotype/sex) were analyzed in the primary screen, and samples from an additional 3–8 mice per group (genotype/sex) were analyzed in follow-up screens.

The observation that homozygous *Cln3* **^Δ^** ^*ex7/8*^ mice displayed consistently increased MCV and increased serum ferritin concentrations, but not alterations in other peripheral blood parameters, including liver enzyme levels, was surprising and prompted us to more carefully analyze the peripheral blood cells and the organs involved in hematopoiesis in homozygous *Cln3* **^Δ^** ^*ex7/8*^ mice. First, we measured the absolute and relative counts of peripheral blood reticulocytes, red blood cell (RBC) precursors that are larger than mature RBCs. These measures were significantly higher for the homozygous *Cln3* **^Δ^** ^*ex7/8*^ mice, compared to those obtained for wild-type and heterozygous littermate mice (p = 0.01, Fig. 5B, C). Further, linear regression analysis of MCV and reticulocyte counts revealed a modest correlation between these two parameters (Fig. 5C), suggesting that the MCV increase in homozygous mutant animals is at least partly explained by the increased reticulocyte count. Other factors such as altered membrane properties in mature RBCs may also account for some of the MCV increase, although no reproducible gross differences in mature RBC morphology were noted between genotypes, and no appreciable autofluorescent or subunit c-positive storage material was detected in the RBCs (data not shown).

**Figure 6:**
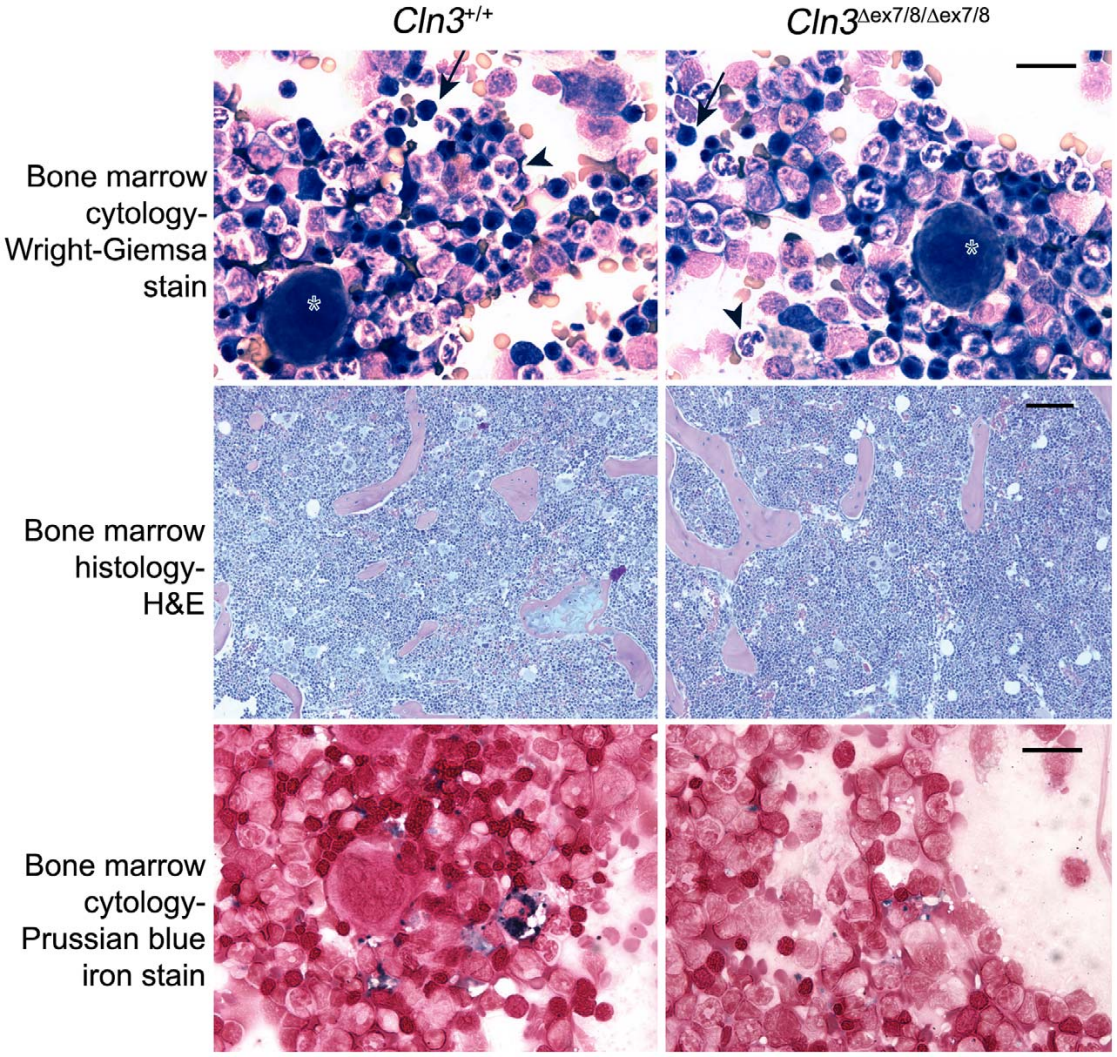
Bone marrow analysis of *Cln3* ^Δ^ ^***ex7/8***^ **mice.** Representative images are shown of Wright-Giemsa-stained bone marrow brush cytology, H&E stained sections of formalin-fixed, paraffin embedded tibias, and iron stained brush cytology, from wild-type (*Cln3^+/+^*) and homozygous mutant (*Cln3* **^Δ^** ^*ex7/8/***Δ***ex7/8*^) mice (n = 3 mice per genotype). Stained iron appears blue. Note the reduced amount of stained iron in *Cln3* **^Δ^** ^*ex7/8/***Δ***ex7/8*^ marrow, compared to wild-type marrow. Arrow, erythroid element; arrowhead, myeloid element; asterisk, megakaryocyte. Scale bars, top and bottom panels = 25 µm; middle panels = 100 µm.

Next, in order to determine whether the increased reticulocyte number was secondary to increased erythroid precursor production in primary sites of hematopoiesis or whether it was due to delayed maturation of erythroid precursors in the periphery, we analyzed liver, spleen, and bone marrow, the major hematopoietic tissues in developing and adult mice. Liver and spleen from homozygous *Cln3* **^Δ^** ^*ex7/8*^ mice were not enlarged or morphologically different from the heterozygous and wild-type littermate tissues (Fig. S9), consistent with previous data [8]. Moreover, brush cytology of bone marrow from wild-type and homozygous *Cln3* **^Δ^** ^*ex7/8*^ mice revealed normal trilineage hematopoiesis and a normal myeloid:erythroid ratio (∼2∶1; Fig. 6). Age-appropriate marrow cellularity (∼80%–85%) and normal hematopoietic architecture were also observed in tibia cross-sections from 12-week-old wild-type and homozygous *Cln3* **^Δ^** ^*ex7/8*^ mice (Fig. 6). Taken together, these data suggest that CLN3 dysfunction affects reticulocyte maturation in the periphery, but does not exert a global effect on primary hematopoiesis. However, we cannot exclude that the grossly normal tissue pathology did not immediately follow a regenerative erythroid response.

Given the consistent observation that homozygous *Cln3* **^Δ^** ^*ex7/8*^ mice had elevated serum ferritin concentrations (Table 3), we also examined iron storage in the set of liver, spleen and bone marrow samples from 12-week-old homozygous and heterozygous *Cln3* **^Δ^** ^*ex7/8*^ mice, and wild-type littermate mice. Ferritin is the major storage protein for intracellular iron, and elevated ferritin concentrations in serum may be an indicator of inflammation or altered iron absorption, utilization or storage in tissues [38]. Staining of the wild-type and homozygous *Cln3* **^Δ^** ^*ex7/8*^ tissues for ferric iron, the major intracellular form of stored iron, showed normal distribution and quantity of iron deposition in the reticuloendothelial system of the red pulp in spleen cross-sections, while minimal to no ferric iron was detected in liver cross-sections for either genotype (Fig. S9). However, robustly stained ferric iron stores were detected in the bone marrow of wild-type mice, primarily in cells that were morphologically consistent with macrophages, while consistently less ferric iron stain was observed in the bone marrow macrophages from homozygous *Cln3* **^Δ^** ^*ex7/8*^ mice (Fig. 6). However, in the peripheral blood and bone marrow, increased numbers of siderocytes and sideroblasts were not observed, suggesting iron utilization in developing erythroid precursors was normal in these mice (data not shown). Thus, in addition to a possible effect on reticulocyte maturation, CLN3 dysfunction in homozygous *Cln3* **^Δ^** ^*ex7/8*^ mice may alter iron homeostasis in bone marrow macrophages. Alternatively, this finding is consistent with utilization of bone marrow iron in the production of reticulocytes, consistent with the increased MCV and reticulocyte counts in these animals. Iron storage, in the form of ferric iron, was also examined in brain sections from 12-week-old wild-type, heterozygous, and homozygous *Cln3* **^Δ^** ^*ex7/8*^ mice, but, notably, no detectable iron stores were observed in brain for any of the mice (data not shown).

**Figure 7:**
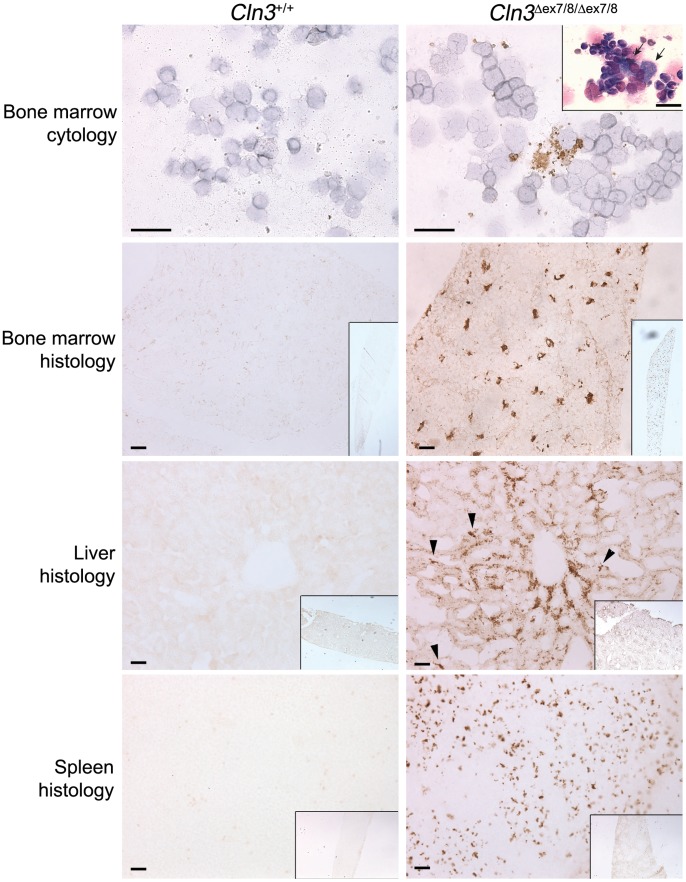
Subunit c immunohistochemistry of major hematopoietic tissues from 12-week-old *Cln3* ^Δ^ ^*ex7/8*^ mice. Representative images from bone marrow brush cytology, tibia cross-sections (‘Bone marrow histology’), and liver and spleen sections immunostained for subunit c are shown for wild-type (*Cln3^+/+^*) and homozygous mutant (*Cln3* **^Δ^** ^*ex7/8/***Δ***ex7/8*^) mice (n = 2−5 mice per tissue/genotype). Inset in *Cln3* **^Δ^** ^*ex7/8/***Δ***ex7/8*^ bone marrow cytology panel (top right panel) shows a sea-blue histiocyte (arrows) from an H&E stained preparation. Sea-blue histiocytes were not found in wild-type bone marrow cytology preparations. Brown stain reflects subunit c-positive storage material, which is most prominent in cells that appear morphologically consistent with macrophages. Arrowheads mark examples of subunit c filled Kupffer cells in liver, also a macrophage lineage cell. Insets in histology panels show lower power magnification of subunit c immunostain. Scale bars = 25 µm.

During our histological analysis of the bone marrow, we also noted the presence of sea-blue histiocytes, macrophages filled with ceroid lipofuscin storage material, in the marrow from homozygous *Cln3* **^Δ^** ^*ex7/8*^ mice (shown in inset of Fig. 7). A similar finding has been reported in bone marrow from JNCL patients [39]. Thus, to more thoroughly determine the extent to which hematopoietic tissues in homozygous *Cln3* **^Δ^** ^*ex7/8*^ mice exhibit the pathologic hallmark of JNCL, storage of mitochondrial subunit c, we analyzed bone marrow, spleen, and liver by subunit c immunohistochemistry. Only faint, punctate staining, likely corresponding to normal endogenous mitochondrial subunit c, was observed in tissues from normal mice. By contrast, consistent with the presence of relatively frequent sea-blue histiocytes in the Wright-Giemsa stained samples, bone marrow from homozygous *Cln3* **^Δ^** ^*ex7/8*^ mice showed a striking accumulation of subunit c in cells that were morphologically consistent with macrophages (Fig. 7). Accumulation of subunit c was also noted in the macrophage-rich red pulp of spleen from homozygous *Cln3* **^Δ^** ^*ex7/8*^ mice, and, as previously described [8], subunit c accumulation was also abundant in liver hepatocytes, particularly those surrounding the central venules of hepatic lobules, as well as in cells likely corresponding to Kupffer cells, liver-resident macrophages (Fig. 7). Thus, subunit c storage is prominent in multiple hematopoietic tissues from homozygous *Cln3* **^Δ^** ^*ex7/8*^ mice, and is particularly abundant in macrophage-lineage cells.

### Vacuolation of Selected Cell Types in Homozygous Cln3**^Δ^** ^ex7/8^ Mice

**Figure 8:**
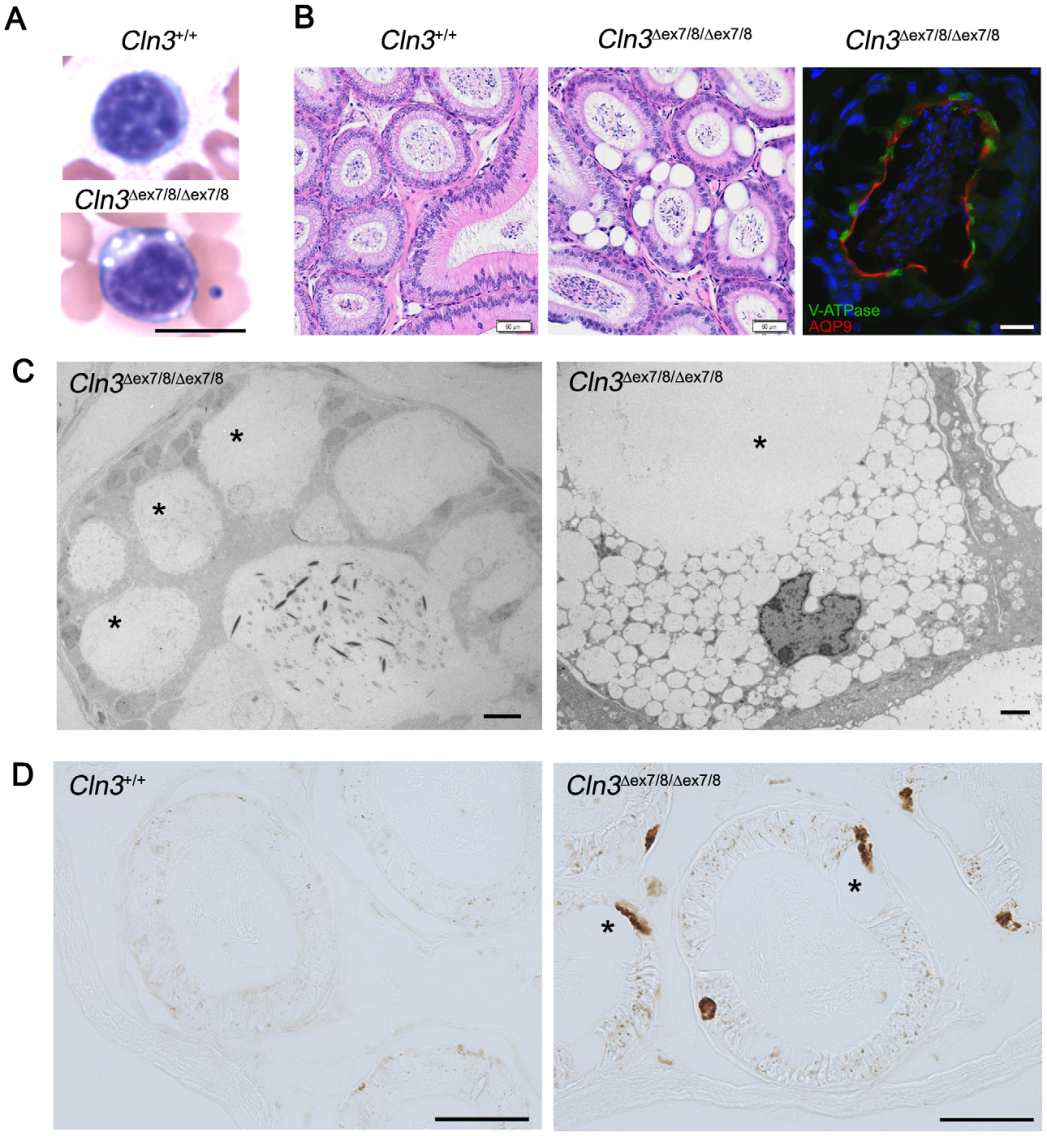
Vacuolation of diverse cell types in homozygous *Cln3* ^Δ^ ^***ex7/8***^ **mice.** (A) Representative images are shown of Wright-Giemsa stained peripheral blood smears from *Cln3^+/+^* and *Cln3* **^Δ^** ^*ex7/8/***Δ***ex7/8*^ littermate mice (scale bar = 10 µm). Note the presence of vacuoles in the cytoplasm of the dark blue stained peripheral blood lymphocyte. (B) Representative images are shown of H&E-stained sections of epididymis from 19-week-old *Cln3^+/+^* and *Cln3* **^Δ^** ^*ex7/8/***Δ***ex7/8*^ littermate male mice (scale bar = 50 µm). A representative image of a section of mutant (*Cln3* **^Δ^** ^*ex7/8/***Δ***ex7/8*^) epididymis immunostained for vacuolar ATPase (V-ATPase, green) and aquaporin-9 (AQP9, red), which highlight the apical (luminal) membrane of clear/narrow cells or principal cells, respectively (scale bar = 25 µm). (C) Representative TEM images of *Cln3* **^Δ^** ^*ex7/8/***Δ***ex7/8*^ epididymis cross-sections are shown. Note both the giant vacuoles and the multiple smaller vacuoles filling the cytoplasm of the clear cells. Also note the relative absence of electron-dense material inside the vacuoles. Scale bars, left panel = 10 µm; right panel = 2 µm. (D) Representative images of subunit c immunostained *Cln3^+/+^* and *Cln3* **^Δ^** ^*ex7/8/***Δ***ex7/8*^ epididymis sections are shown. Asterisks (*) mark some of the large vacuoles. Scale bars = 50 µm. Blood smears and epididymides from at least 10 mice per genotype were analyzed in total, and abnormal vacuolation was observed in all of the *Cln3* **^Δ^** ^*ex7/8/***Δ***ex7/8*^ mice and in none of the wild-type or *Cln3^+/^* ^**Δ***ex7/8*^ mice.

Evaluation of peripheral blood smears for vacuolated lymphocytes is a useful diagnostic tool in the workup of JNCL patients [40], [41]. In this study, we similarly detected abnormal vacuolation in ∼5–15% of the peripheral blood lymphocytes from homozygous *Cln3* **^Δ^** ^*ex7/8*^ mice, where the proportion of lymphocytes with a vacuolated appearance tended to increase with age (8.3±1 at postnatal day 7, versus 14.8±2 at 16 weeks of age). The numbers of vacuolated lymphocytes in wild-type and heterozygous littermate mice were typically <5% of the total counted lymphocytes (Fig. 8A).

Intriguingly, we also discovered a profound vacuolation in cells of the male reproductive tract that was absent in all wild-type and heterozygous *Cln3* **^Δ^** ^*ex7/8*^ mice examined (Fig. 8B). The epididymis, an organ with a key role in sperm maturation and male fertility, is a narrow, tightly coiled tube connecting the efferent ducts to the vas deferens. Marker staining revealed that the cells bearing the giant vacuoles were the well-studied V-ATPase-expressing narrow and clear cells, while aquaporin-9-expressing principal cells [42] showed relatively normal morphology in homozygous *Cln3* **^Δ^** ^*ex7/8*^ mice. Transmission electron microscopic (TEM) analysis confirmed the presence of massive, mostly translucent vacuoles in the clear cells, and further revealed an accumulation of smaller vacuoles in many of the cells as well (Fig. 8C). Neither Oil Red O nor Periodic Acid Schiff (PAS) stained the intravacuolar material, indicating that lipids and glycogen, respectively, were not a major component (data not shown). Autofluorescence analysis and subunit c immunostaining also did not highlight the inside of the vacuoles, consistent with lack of electron-dense storage material inside the vacuoles by TEM analysis. However, large extra-vacuolar deposits were often observed in the severely misshapen clear cells (Fig. 8D). Notably, autofluorescent, subunit c-positive storage material was also detected in the principal cells, appearing as smaller puncta compared to the large deposits associated with the vacuolated cells (Fig. 8D). Remarkably, despite the presence of these giant vacuoles, male homozygous *Cln3* **^Δ^** ^*ex7/8*^ mice on the C57BL/6N background were able to successfully breed, at least to 20 weeks of age (data not shown), consistent with previously reported observations [8].

**Figure 9:**
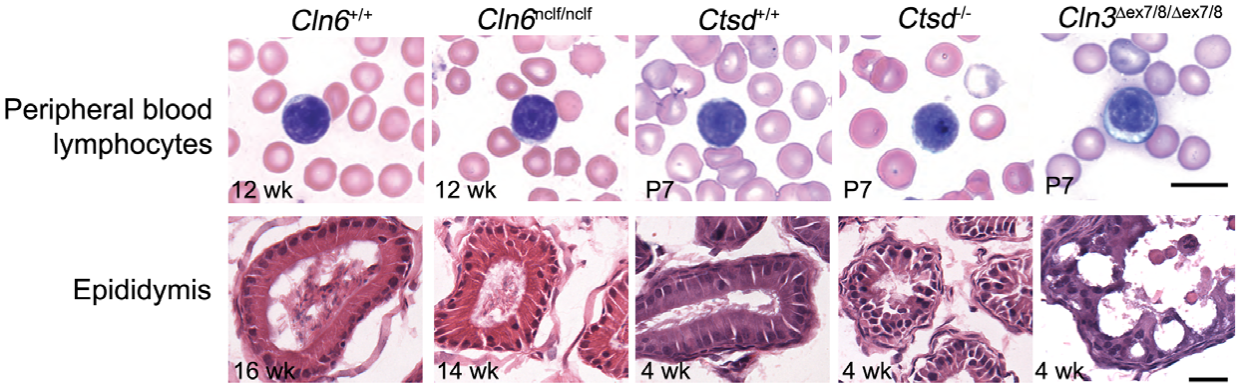
Cellular vacuolation is specific to CLN3 dysfunction. Representative images are shown of Wright-Giemsa stained peripheral blood smears and H&E-stained sections of male epididymis from mice representing three different forms of NCL. Ages of the mice at the time of sample collection are indicated. Note the obvious vacuolation of peripheral blood lymphocytes and clear cells of the epididymis in young homozygous *Cln3* **^Δ^** ^*ex7/8*^ mice (*Cln3* **^Δ^** ^*ex7/8/***Δ***ex7/8*^), shown at postnatal day 7 (P7) and 4 weeks of age (4 wk), respectively. Note the lack of abnormal vacuoles in *Cln6^nclf/nclf^* and *Ctsd^−/−^* mice, which are models of variant late-infantile and congenital NCL, respectively. N = 3−4 mice per genotype. Scale bars, top panels = 10 µm; bottom panels = 25 µm.

Lymphocyte vacuolation, among the NCLs, is thought to be diagnostic for *CLN3* mutation [40]. Therefore, we sought to determine whether the vacuolation phenotypes uncovered in this study were also unique to *Cln3* **^Δ^** ^*ex7/8*^ mice, versus mouse models for two other forms of NCL. Peripheral blood smears were prepared from 12-week old *Cln6^nclf^* mice, which model a variant late-infantile form of NCL (CLN6, MIM#601780) [43], [44], [45], and from postnatal day 7 (P7) *Ctsd* knock-out mice, which model the most severe form of NCL (congenital NCL; CLN10, MIM#610127) [46], [47]. Unlike the vacuolated appearance of the lymphocytes from 12-week-old and P7 homozygous *Cln3* **^Δ^** ^*ex7/8*^ mice (Figs. 8A, 9), there were no morphological differences in homozygous *Cln6^nclf/nclf^* or in *Ctsd^−/−^* mouse peripheral blood lymphocytes (Fig. 9). Notably, however, we did observe a significant reduction in numbers of peripheral blood lymphocytes in *Ctsd^−/−^* mice (∼50% reduced from age-matched control mice, as assessed by visual inspection of peripheral blood smears). This is consistent with a previously reported finding of progressive lymphopenia in the thymus and spleen of *Ctsd^−/−^* mice [46]. Similar to our lymphocyte analysis, we also found no evidence for morphological differences in the epididymal clear cells from homozygous *Cln6^nclf^* or *Ctsd* knock-out male mice, as compared to wild-type littermate mice (Fig. 9).

Taken together, these data suggest that CLN3 dysfunction specifically leads to an abnormal vacuolation across selected, apparently diverse subsets of cells, which is not directly related to the classical lysosomal accumulations in JNCL. Further study of abnormalities in the peripheral blood and in the epididymis of homozygous *Cln3* **^Δ^** ^*ex7/8*^ mice may provide important new clues to understanding CLN3 function.

## Discussion

In this study, we have broadly probed for early phenotypes in the previously established *Cln3* **^Δ^** ^*ex7/8*^ knock-in mouse model of JNCL, now on an inbred C57BL/6N genetic background. Our results suggest that the common JNCL mutation, which is recapitulated in these mice, leads to early onset sensorimotor processing abnormalities that long precede neuronal cell loss, and we have documented clear evidence of later onset retinal functional decline in homozygous *Cln3* **^Δ^** ^*ex7/8*^ mice. Our data also strongly support roles for CLN3 in hematopoiesis and epididymal biology, and possibly in metabolic regulation, which have not previously been recognized. Intriguingly, our study also uncovered possible differences in disease manifestations in males versus females, consistent with emerging patient data suggesting sex influences JNCL disease course [48].

The results of the neurological analysis of *Cln3* **^Δ^** ^*ex7/8*^ mice in this and other studies [8], [27] together suggest a battery of mouse behavioral assays that may be useful in future pharmacologic or genetic disease modifier studies in search of effective JNCL treatments. However, inconsistencies between our results here and the results of behavioral studies performed on *Cln3* **^Δ^** ^*ex7/8*^ mice by other groups [27], [49] also clearly indicate the need for standardized mouse behavioral testing paradigms. The differences in the performance of *Cln3* **^Δ^** ^*ex7/8*^ mice in rotarod and open field assays across this study and the studies described in other reports may be attributable to equipment differences, methodology, data analysis, testing age, genetic background, and/or environmental influences. Nevertheless, other tests performed in this study and others, such as the pole-climbing test and gait analysis [8], remain consistent with a decline in neuromotor performance in homozygous *Cln3* **^Δ^** ^*ex7/8*^ mice.

The observation in this study that male homozygous *Cln3* **^Δ^** ^*ex7/8*^ mice displayed reduced PPI of the acoustic startle response and that female homozygous *Cln3* **^Δ^** ^*ex7/8*^ mice displayed slightly reduced thermal nociception was intriguing. Reduced thermal nociception has also been reported in a study of *Clcn6* knock-out female mice, which also accumulate subunit c of the mitochondrial ATPase, the hallmark NCL storage material [50]. Deficits in PPI are common in patients with neuropsychiatric diseases, including schizophrenia, Huntington’s disease and Parkinson’s disease, and in other seizure disorders [51], [52], [53]. JNCL patients also often suffer from psychiatric symptoms, including depression, anxiety and auditory and/or visual hallucinations [41], [54], [55]. Pain assessment and PPI testing of JNCL patients may be warranted, as these tests, if revealing of phenotypes, could be a useful means of further monitoring disease progression, alongside other already established clinical tests [56], [57].

While we did not find a clear neuropathological correlate to the sensory and motor functional deficits in the young adult mice studied here, previous studies in older homozygous *Cln3* **^Δ^** ^*ex7/8*^ mice, albeit on a different genetic background, have demonstrated neuronal cell loss specifically within the thalamus and cortex [25]. The thalamus relays and processes sensory information in circuits that communicate with neurons of the cerebral cortex. It is therefore tempting to speculate that the functional deficits measured here in homozygous *Cln3* **^Δ^** ^*ex7/8*^ mice may reflect early dysfunction in the neurons that later die within the thalamus and cortex. For example, synaptic dysfunction in these brain regions may precede neuronal cell dropout. Indeed, early synaptic defects have been documented in other mouse models of NCL [58], [59], [60], [61], [62], and CLN3 is present in vesicular compartments at neuronal synapses [63]. However, more careful chronological analyses pairing additional sensorimotor functional tests with neuropathological studies are needed to fully delineate the complex events involved in the neurological disease in homozygous *Cln3* **^Δ^** ^*ex7/8*^ mice on the C57BL/6N genetic background. This knowledge will facilitate improved mouse testing protocols for emerging disease modifier studies, which will be a key factor in determining efficacy of possible treatments.

Vision loss is typically the presenting symptom among JNCL patients [41], [57]. It was therefore of considerable interest to determine more precisely whether homozygous *Cln3* **^Δ^** ^*ex7/8*^ mice recapitulate this feature of the human disease. The apparently selective loss of the b-wave amplitude, with nearly normal a-wave amplitude in homozygous *Cln3* **^Δ^** ^*ex7/8*^ mice, as compared to littermate control mice, strongly suggests that there is a defect in synaptic transmission or a loss of function in the bipolar cells of the inner nuclear layer of the retina in homozygous *Cln3* **^Δ^** ^*ex7/8*^ mice. These data are in accordance with the retinal findings in *Cln3* knock-out mice reported by Katz et al. [64] and with the attenuation of optic nerve function in *Cln3*-knock-out mice reported by Weimer et al., which may occur secondarily due to a defect in the second order retinal neurons [65]. In JNCL patients, the ERG is typically flat by the time of clinical presentation. To our knowledge, there is only one report describing early ERG changes in human patients [66]. In two patients of about 2 years of age, Weleber et al observed a selective decline of the scotopic b-wave [66]. Therefore, while the onset timing of the retinal function decline in homozygous *Cln3* **^Δ^** ^*ex7/8*^ mice, like in the other *Cln3* mouse models, may not coincide with that observed in JNCL patients, there is accumulating evidence that the *Cln3* mouse models indeed recapitulate the early features of retinal degeneration in JNCL, and that photoreceptor loss in human patients, which occurs at a much later stage in the mouse models, might result from degenerative processes of the middle and/or inner retina. We note that recently, Mattapallil et al reported the existence of the *Crb1^rd8^* mutation in the C57BL/6N genetic background, which could confound retinal function studies in transgenic mouse models established on this background [67]. In *Crb1^rd8/rd8^* mice, there is an abnormal fundus with retinal dysplasia, but the ERG remains normal, except for a slight reduction in the V_max_ of the dark-adapted b-wave at greater than 9 months of age. The light-adapted ERG, which was the earliest defect seen in homozygous *Cln3* **^Δ^** ^*ex7/8*^ mice in our study, is reported to be unchanged in homozygous *Crb1^rd8^* mice [68]. Therefore, while the mice in our study were not genotyped for the *Crb1^rd8^* mutation, given the timing of our study relative to the recent report of its prevalence in the C57BL/6N strain, it appears that the retinal degeneration phenotypes found in the homozygous *Cln3* **^Δ^** ^*ex7/8*^ mice studied here are distinct from the *Crb1^rd8/rd8^* phenotype. However, it is possible that the *Crb1^rd8^* mutation, if present, could sensitize to retinal dysfunction due to the *Cln3* **^Δ^** ^*ex7/8*^ homozygous mutation. Further studies will be needed to resolve this question.

In addition to expanding our knowledge of CNS phenotypes in this accurate genetic model of JNCL, our broader screen described here has also identified extra-CNS abnormalities. Subtle differences were observed in the metabolism, cardiovascular, immunology, and clinical chemistry screens of young adult *Cln3* **^Δ^** ^*ex7/8*^ mice, indicating that further studies of these defects in this and other CLN3-deficiency models could provide important new insights into CLN3 protein function. Though no evidence was found for cardiovascular defects in young adult homozygous *Cln3* **^Δ^** ^*ex7/8*^ mice in this study, it remains a possibility that defects could arise at later ages, particularly given that the cardiovascular system defects reported in JNCL patients were relatively late-onset [3], [4].

The subtle metabolic changes found in *Cln3* **^Δ^** ^*ex7/8*^ mice, which were maintained on normal fat chow (5%) in this study, may be indicative of a role for CLN3 in the regulatory pathways that control metabolism or directly in peripheral tissues. Moreover, it was intriguing that one copy of the *Cln3* **^Δ^** ^*ex7/8*^ mutation appeared to be sufficient to alter resting body temperature and minimum oxygen consumption, suggesting that in this process, there may be a specific threshold for normal CLN3 activity required to maintain normal metabolic function. While the vast majority of phenotypes uncovered in our comprehensive screen were recessively inherited, consistent with JNCL disease genetics, the observation that heterozygous *Cln3* **^Δ^** ^*ex7/8*^ mutation can influence more subtle parameters is also important, as this knowledge will likely improve our broader understanding of CLN3 biology and will be useful in disease biomarker development. Further studies in which diet challenges alter the metabolic pathways in *Cln3* **^Δ^** ^*ex7/8*^ mice are likely to substantially improve our understanding of this potential aspect of CLN3 deficiency. Intriguingly, metabolic changes have also been reported in mouse models of other forms of NCL [69], implying metabolism may be an under-recognized player in multiple forms of NCL.

It was of particular interest to us that the analyses of peripheral blood from young adult homozygous *Cln3* **^Δ^** ^*ex7/8*^ mice consistently highlighted alterations in MCV and in serum ferritin concentrations. Our follow-up studies indicated that the increased MCV, which our data suggests is reflective of increased reticulocytes in peripheral blood, may signal abnormalities in hematopoiesis, specifically in homozygous *Cln3* **^Δ^** ^*ex7/8*^ mice. Given the apparently normal erythroid precursor production in primary sites of hematopoiesis in mutant mice, we hypothesize that the increased reticulocyte count in mutant animals may be at least partly due to delayed peripheral maturation of erythroid precursors, a hypothesis further motivated by 1) the observation that impaired autophagy in *Atg7* ^−/−^ mice affects reticulocyte maturation by delaying mitochondrial clearance through a selective process known as mitophagy [70], 2) that CLN3 deficiency causes defects in autophagy [71], and 3) that CLN3 was shown to participate in a protein complex with Atg7 in a proteomic analysis of the autophagy interactome in 293T cells [72]. As the mechanistic details for increased MCV and reticulocyte counts in our JNCL model become clearer, these two parameters may eventually serve as peripheral biomarkers of autophagic function in JNCL. Interestingly, autophagy is also required for the development and survival of lymphocytes, including T cells [73]. Whether a defect in autophagy underlies the relative T-cell deficiency in homozygous *Cln3* **^Δ^** ^*ex7/8*^ male mice remains a subject for further investigation. Changes in T-cell frequency, for example in the CD4/CD8 ratio, may also be indicative of an ongoing inflammation in these mice. Therefore, additional studies of these blood parameters, particularly in JNCL patients, may be useful in gaining an improved understanding of the involvement of the immune system in the disease process.

The elevated serum ferritin we observed in homozygous *Cln3* **^Δ^** ^*ex7/8*^ mice, and decreased storage of iron in bone marrow macrophages, may or may not be related. Serum ferritin concentrations may correlate with total body iron stores. However, serum iron levels were not different in homozygous *Cln3* **^Δ^** ^*ex7/8*^ mice, and stored iron in the tissues examined was different only in bone marrow macrophages, where it was reduced in homozygous *Cln3* **^Δ^** ^*ex7/8*^ mice relative to wild-type littermate mice. This apparent discordance between the two related parameters was somewhat surprising, though other mouse studies have made similar observations, demonstrating the complexity of iron homeostasis [74].

Interestingly, autophagy and phagocytosis, which have overlapping mechanisms, play important roles in iron homeostasis [reviewed in [38]]. In erythrophagocytosis, in which macrophages phagocytose damaged or senescent red blood cells, catabolize hemoglobin, and liberate and store iron from heme, mediators of autophagy including LC3 and ATG12 are thought to play an important role [reviewed in [75]]. Ferritin turnover is also at least partially mediated via autophagy, as its half-life is increased in *Atg5*- and *Atg7*-deficienct cells and in cells treated with the vacuolar ATPase inhibitor bafilomycin A1 [76]. Therefore, defects in autophagy and/or phagocytosis may play a causal role in the rather specific storage of subunit c in cells of the macrophage lineage, the decreased ferric iron in bone marrow macrophages, and the elevated serum ferritin in homozygous *Cln3* **^Δ^** ^*ex7/8*^ mice. Although splenic red pulp from homozygous *Cln3* **^Δ^** ^*ex7/8*^ mice showed no gross differences in iron storage compared to wild-type mice, subtle differences at the level of individual tissue macrophages may be difficult to appreciate in standard histological preparations. The complex regulation of ferritin and iron homeostasis also involves specialized trafficking pathways within the endosomal-lysosomal system, and these pathways are likely to be modified by inflammatory processes. Thus, the elevated serum ferritin concentrations and reduced iron storage in bone marrow macrophages from homozygous *Cln3* **^Δ^** ^*ex7/8*^ mice may occur secondarily to a number of biological pathways disrupted by CLN3 dysfunction. Further studies are required in order to fully understand the causes and biological significance of these findings. Nevertheless, as with the altered T cell frequencies and the increased MCV and reticulocyte counts, the novel findings related to iron homeostasis suggest new possible biomarkers that should be further studied in both *Cln3* **^Δ^** ^*ex7/8*^ mice and in JNCL patients.

The vacuolation phenotypes seen in genetically accurate *Cln3* **^Δ^** ^*ex7/8*^ mice are reminiscent of those seen in other lysosomal storage diseases, though intriguingly not in other forms of NCL. Vacuolated lymphocytes, although often heterogeneous in number and vacuolar size, are a useful screening tool for JNCL, sialic acid storage disease, mannosidosis, and Pompe’s disease, among others [77]. Our study suggested an increase in the size and frequency of lymphocytic vacuoles between P7 and 12 weeks of age in homozygous *Cln3* **^Δ^** ^*ex7/8*^ mice. However, the progression of this phenotype with age in JNCL patients has not yet been fully evaluated, nor has its correlation with *CLN3* genotypes or clinical severity. We propose this should be a priority in JNCL biomarker development.

Murine models of the related G_M2_-ganglioside storage disorders, Tay-Sachs and Sandhoff disease, in which the *Hexa* and *Hexb* genes are disrupted, respectively, have been reported to display marked accumulation of vacuoles in cells of the epididymis [78], [79], which appear strikingly similar to those we observed in our JNCL model. Further studies will be necessary to fully elucidate the identity and biochemical properties of these vacuolar structures as well as their effect on epididymal physiology. However, it is intriguing that homozygous *Cln3* **^Δ^** ^*ex7/8*^ mice also display neuronal accumulations of G_M2_-ganglioside (unpublished data, personal communication Drs. Stephen Walkley and Matthew Micsenyi), further supporting overlapping pathophysiology between JNCL and Tay-Sachs and Sandhoff diseases.

In summary, this large-scale phenotyping study of *Cln3* **^Δ^** ^*ex7/8*^ mice has identified a number of possible new biomarkers that merit further study, and we have more thoroughly defined aspects of the JNCL-like disease in these mice. The new information reported here substantially broadens the possible biological processes in which CLN3 may play a role, establishing new systems in which to study CLN3 function and dysfunction.

## Materials and Methods

### Ethics Statement

This mouse work was in accordance with the National Institutes of Health Guide for the Care and Use of Laboratory Animals. This study was reviewed and approved by the Massachusetts General Hospital (MGH) Subcommittee of Research Animal Care (SRAC), which serves as the Institutional Animal Care and Use Committee (IACUC) for MGH (Protocol #2008N000013); the study was also reviewed and approved to be in accordance with German legal guidelines and by authority of the Regierung von Oberbayern.

### Cln3**^Δ^** ^ex7/8^ Mice

Young adult *Cln3* **^Δ^** ^ex7/8^ mice congenic on the C57BL/6N genetic background (120 total mice = 41 homozygous *Cln3* **^Δ^** ^*ex7/8*^ mice, 39 heterozygous *Cln3* **^Δ^** ^*ex7/8*^ mice, and 40 wild-type littermates) were tested in a large-scale phenotyping workflow established by the German Mouse Clinic [www.mouseclinic.de] [28], [29], [30]. The generation of the *Cln3* **^Δ^** ^ex7/8^ allele has been described previously [8]. To move the *Cln3* **^Δ^** ^*ex7/8*^ allele onto the C57BL/6N genetic background, heterozygous *Cln3* **^Δ^** ^*ex7/8*^ CD1 mice were backcrossed to wild-type C57BL/6N (C57BL/6NCrl, Charles River Labs, strain code = 027) for more than 15 generations. SNP genotyping [80], [81] with a panel of markers that could discriminate C57BL/6N from 129 mice (Illumina custom 768 SNP panel) through the Harvard Medical School-Partners Healthcare Center for Genetics and Genomics (HPCGG) (http://pcpgm.partners.org/research-services/genotyping) was performed at approximately the N12 generation. All SNP markers were homozygous for C57BL/6N, with the exception of a few markers immediately near the *Cln3* locus, which were 129 from the initial generation of the allele [8] [data not shown]. Heterozygote x heterozygote intercrosses with animals from generations N17 and N18 were set up at Massachusetts General Hospital (MGH) in order to generate the experimental mice for the primary screen, which were shipped to the German Mouse Clinic (GMC) at 6 weeks of age. Upon arrival at the GMC facility, mice were acclimated for four weeks in a holding room prior to the initiation of any experiments. Two different pipelines of mice were sent to the GMC for the study. Pipeline 1 underwent screening in components of the Dysmorphology, Cardiovascular, Metabolism, Clinical Chemistry, Eye, Lung, and Molecular Phenotyping Modules [www.mouseclinic.de]. Pipeline 2 underwent screening in components of the Neurology, Behavior, Nociception, Clinical Chemistry, Immunology, Allergy, Cardiovascular, Steroid, and Pathology Modules [www.mouseclinic.de]. For follow-up studies, additional cohorts of mice from the congenic C57BL/6N *Cln3* **^Δ^** ^*ex7/8*^ line were generated and analyzed at MGH, or for electroretinography, at Charité-Eye Hospital, Campus Virchow-Klinikum, in Berlin, Germany.

Breeder *Cln6^nclf^* mice, which have been previously described [43], were obtained from The Jackson Laboratory (Stock #003605, www.jax.org). Breeders were used to establish a mouse colony at MGH, maintained on the C57BL/6J background by crossing *Cln6^nclf^* heterozygotes to C57BL/6J wild-type mice. Breeder *Ctsd* knock-out mice, also previously described [46], were a generous gift from Dr. Paul Saftig (University of Kiel), and breeders were used to establish a mouse colony at MGH, maintained on the C57BL/6J background by crossing *Ctsd^+/−^* mice to C57BL/6J wild-type mice. Importantly, for comparative studies between *Cln3* **^Δ^** ^*ex7/8*^ mice and the *Cln6^nclf^* and *Ctsd* knock-out mice, we also backcrossed *Cln3* **^Δ^** ^*ex7/8*^ mice from the C57BL/6N genetic background to the C57BL/6J background, which though highly related to C57BL/6N [80], is known to display some behavioral differences [82]. No differences were observed in the blood parameter and epididymal phenotypes in homozygous *Cln3* **^Δ^** ^*ex7/8*^ mice on the C57BL/6N versus C57BL/6J genetic backgrounds (data not shown). The C57BL/6J congenic *Cln3* **^Δ^** ^*ex7/8*^ mice have been made available at The Jackson Laboratory Repository (Stock # 017895, www.jax.org).

### Open Field and Prepulse Inhibition of the Acoustic Startle Reflex

The Open Field test and Prepulse Inhibition (PPI) of the Acoustic Startle Reflex (ASR) were assessed according to the standardized phenotyping screens developed by the Eumorphia partners [83], available as EMPReSSslim protocols (see www.eumodic.org). The Open Field apparatus consisted of a transparent and infrared light permeable acrylic test arena with a smooth floor (internal measurements: 45.5×45.5.×39.5 cm). Illumination levels were set at approximately 150 lux in the corners and 200 lux in the middle of the test arena. Data were recorded and analyzed using the ActiMot system (TSE, Bad Homburg, Germany). The ASR/PPI protocol was adapted to the specifications of our startle equipment (Med Associates Inc., VT, USA). Background noise (NS = no stimulus) was 65 db and trial types for ASR included 7 different stimulus intensities (NS, 70, 80, 90, 100, 110, 120 db). Trial types for PPI included 4 different prepulse intensities (67, 69, 73, 81 db), and each prepulse preceded the startle pulse (110 db) by a 50 milliseconds inter-stimulus interval. 9–10 mice per group (genotype/sex) were analyzed.

### Modified SHIRPA Protocol and Rotarod Analysis

The modified SHIRPA protocol and the rotarod analysis were performed according to standardized protocols [www.eumodic.org; [84]]. For rotarod analysis, 3 trials with 15 minutes inter-trial-intervals were performed on an accelerating rotarod (4–40 rpm/5 min; Bioseb, France). For statistical analysis, a Chi-square test for categorical data was used for SHIRPA analysis data, and a linear mixed effects model including sex, genotype, trial number and body mass was used for metric data from rotarod analysis (Software: S-Plus, Insightful). Data were grouped by sex and genotype of the mice, which were generated from 9–10 mice per group.

### Pole Test

For the pole test, mice were placed in a head-up position at the top of a vertical bar (50 cm). On two consecutive days prior to testing, mice were habituated to the apparatus. For testing, the time until the mouse turned around to climb down (t-turn) and the total time until the mouse reached the floor with its forepaws (t-total) were each recorded. Maximum time for the testing period allowed was 120 seconds. The test was repeated five times, and for each session of five descents, the best performance was kept for t-turn and t-total. Data were grouped by sex and genotype of the mice, which were generated from 9–10 mice per group. Pole time results were analyzed for sex and genotype effects using a linear model.

### Statistical Analysis of Neurological/behavioral Data

Statistical analysis was performed using a statistical package Statgraphics^®^ (Statistical Graphics Corporation, Rockville, MD). Differences between the groups were statistically tested with a two-factorial ANOVA. A post-hoc test was performed with a least significant difference (LSD) test (Tukey). Statistical significance was assumed at p<0.05.

### Nociception Hot-plate Test

The nociception hot-plate test used here has been previously described [30]. Briefly, mice were placed on a metal surface maintained at 52±0.2°C [Hot plate system from TSE GMBH, Germany; [85]]. Locomotion of the mouse on the hot plate was constrained to a circular area with a diameter of 28 cm using a 20-cm high Plexiglass wall. Mice remained on the plate until they performed one of three behaviors that were regarded as indicative of nociception: hind paw lick, hind paw shake/flutter, or jumping. We evaluated only the hind paw responses, since fore paw licking and lifting are components of normal grooming behavior. Each mouse was tested only once since repeated testing leads to profound changes in response latencies. The latency was recorded to the nearest 0.1 second. To avoid tissue injury, a 30-second cut-off time was applied. Data were grouped by sex and genotype of the mice, which were generated from 9–10 mice per group.

### Funduscopy, Slit Lamp Biomicroscopy, and Laser Interference Biometry

The posterior parts of both eyes were examined by funduscopy. After pupil dilation with one drop of 1% atropine, the mouse was grasped firmly in one hand and clinically evaluated using a head-worn indirect ophthalmoscope (Sigma 150 K, Heine Optotechnik, Herrsching, Germany) in conjunction with a condensing lens (90 D lens, Volk, Mentor, OH, USA) mounted between the ophthalmoscope and the eye.

Mice were examined biomicroscopically for eye abnormalities as previously described [86]. Briefly, pupils were dilated with a 1% atropine solution applied to the eyes at least 10 min prior to examination. Both eyes of the mice were examined by slit lamp biomicroscopy (Zeiss SLM30) at 48× magnification with a narrow beam slit lamp illumination at a 25–30° angle from the direction of observation. Observed phenotypic variants of the eyes were carefully documented.

Laser Interference Biometry (LIB) was performed using the “ACMaster” (Meditec, Carl Zeiss, Jena, Germany) equipped with a new technique, optical low coherence interferometry (OLCI), adapted for short measurement distances [87]. Mice were anaesthetized with 137 mg Ketamine and 6.6 mg Xylazine per kg body weight and placed in front of the ACMaster. Laser interference biometry data were statistically analyzed using MS-Excel. Data were grouped by sex and genotype of the mice, which were generated from 9–10 mice per group. Differences between mouse groups were evaluated with the Student’s t-test. Statistical significance was assumed at p<0.05.

### Electroretinography

Electroretinography (ERG) studies were performed on a different cohort of mice than those that were part of the primary screen performed at the GMC. Eight homozygous *Cln3* **^Δ^** ^ex7/8^ mice and seven wild-type mice were examined by ERG, at the ages of 5, 9 and 16 months. Prior to ERG recordings the mice were kept in darkness for at least 2 hours. Pupils were dilated by application of 0.5% tropicamide and 1% atropine. Xylazine (20 mg/kg body weight) and ketamine (40 mg/kg) were injected subcutaneously to anesthetize the mice prior to placement of electrodes. A monopolar contact lens electrode served as the recording electrode. Silver needle electrodes fixed subcutaneously served as reference and neutral electrodes. While the ERG was recorded, the mouse was placed into a Ganzfeld bowl (Toennies Multiliner Vision, Höchberg, Germany), oriented so that the examined eye faced the back of the globe. The background light for recording cone responses was calibrated using a Minolta Spot–Luminance-Meter (30 cdm^−2^). For data analysis and presentation, the ERG recordings within the same genotype group were averaged. ERG recordings from males and females of like genotype were pooled for the data analysis. For statistical analysis, an unpaired, one-tailed Student’s t-test was used, assuming decreased amplitudes in homozygous *Cln3* **^Δ^** ^ex7/8^ mice.

### Tail-cuff Blood Pressure Measurement

Blood pressure was measured in unanesthetized mice with a non-invasive tail-cuff method using the MC4000 Blood Pressure Analysis Systems (Hatteras Instruments Inc., Cary, North Carolina, USA). Four animals at a time were restrained on a pre-warmed metal platform in metal boxes. The tails were looped through a tail-cuff and fixed in a notch containing an optical path equipped with an LED light and a photosensor. The blood pulse wave in the tail artery is transformed into an optical pulse signal by measurement of light extinction. Pulse detection, cuff inflation and pressure evaluation were automated by the system software. After five initial inflation runs for habituation, 12 measurement runs were performed for each animal per session. Runs with movement artifacts were excluded from the dataset used for further analysis. Mice were habituated to the apparatus and protocol one day prior to the testing period, which entailed taking measurements on four consecutive days between 8∶30 and 11∶30 AM. For blood pressure analysis, at least 20 to 60 individual measurements were pooled to obtain a mean over the four measurement days for each animal. 5–10 mice per group (genotype/sex) were analyzed.

### Nt-proANP Analysis

Since ANP is rapidly cleared from the circulation (half-life 3–4 minutes), the more stable N-terminal propeptide (Nt-proANP (1-98), half-life 60–120 minutes), which is cleaved from the released pro-hormone, was used to more accurately reflect chronic levels of ANP secretion [88], [89].

Blood samples were taken by puncturing the retro-orbital sinus of mice under sedation with isofluorane. Blood was collected into lithium-heparin coated tubes and plasma was separated from cells by centrifugation.

Plasma Nt-proANP concentrations were quantified using a commercial Nt-proANP enzyme-linked immunosorbent assay (Biomedica Medizinprodukte, Vienna, Austria). The microtiter plates were read in a Tecan GENios Pro Plate Reader (Tecan Deutschland GmbH, Crailsheim, Germany), controlled by the Magellan Software package (Version V 5.03; Tecan). Optical density (OD) was measured at a wavelength of 450 nm, and additionally as reference wavelength of 612 nm. Detection limit of the assay was 0.05 nmol/l, and within- and between-run coefficients of variation were 2% and 4%, respectively. Samples from 9–10 mice per group (genotype/sex) were analyzed.

### Echocardiography

Left ventricular function was determined using a small animal ultrasound biomicroscope with a 30-MHz transducer and 30-Hz frame rate (Vevo 660; VisualSonics, Toronto, Ontario). The shaved and anesthetized mice (1% isoflurane inhalation, Baxter, Munich, Germany) were fixed in supine position on a heated platform equipped with ECG electrodes for heart rate monitoring. Body temperature was maintained at 36–38°C, monitored via a rectal thermometer (Indus Instruments, Houston, Texas, USA). Left ventricular parasternal short-axis views were obtained at the papillary muscle level to record 2-dimensional B-mode images and time-motion M-mode images. In the M-mode imaging, we performed three recordings per animal and averaged measurements from four cardiac cycles of each record for the left ventricular internal diameter in diastole (LVID dia) and the left ventricular internal diameter in systole (LVID sys). Markers were set using the leading-edge convention, as suggested by the American Society of Echocardiography [90]. From these measurements several parameter were derived as described by [91]:

Fractional Shortening = [(LVID dia-LVID sys)/LVID dia]*100.

Left ventricular volume in diastole = [7/(2.4+LVID dia)]*(LVID dia)^3^.

Left ventricular volume in systole = [7/(2.4+LVID sys)]*(LVID sys)^3^.

Ejection Fraction = [(LV volume dia - LV volume sys)/LV volume dia]*100.

In addition the heart rate was determined graphically using the contraction intervals in the area of measurements. In the quantitative ECG analysis, sets of five analyzed beats were averaged for one animal. A total of 6–8 mice per group (genotype/sex) were analyzed.

### Analysis of Data from Cardiovascular Screen

In general, the data were statistically analyzed using Statistica. A two-factorial ANOVA was used for an analysis of group differences between levels of sex and genotype. Post-hoc analysis for multiple comparisons included a Duncan’s Multiple Range Test.

### Immunology

The flow cytometric analysis of leukocyte populations in peripheral blood, from 9–10 mice per group (genotype/sex), was based on two 10-parameter staining panels, as described in Gailus-Durner *et al.*, 2009 [92], covering markers for B cells (CD19, IgD, B220), T cells (CD3, CD4, CD8, CD5), granulocytes (GR-1, CD11b), NK cells (NKp46) and further subsets (CD44, CD62L, CD25, Ly6C).

The preparation of the cells was performed as described [30]. In short, after red blood cell lysis (H_2_O, NH_4_Cl, Tris–HCl, pH = 7.5), cells were washed in FACS buffer (PBS, 0.5% BSA, 0.02% sodium azide, pH 7.45) and incubated with Fc block (anti-mouse CD16/32), fluorescence-conjugated antibodies (BD Biosciences, Heidelberg, Germany) and propidium iodide. Approximately 30,000 leukocytes per sample were acquired with a FACS LSR II HTS (BD, San Diego, USA). Analysis, including statistical analysis, was performed with FlowJo (Tree Star, Inc., Oregon USA); dead cells were eliminated on the basis of their propidium iodide signal and gating for leukocytes (CD45+) and subsequent subsets.

The frequencies of leukocyte subsets were determined as a percentage of leukocytes (CD45+) or the respective parent gate (CD8+ or CD4+ T cells, in case of Ly6C+ cells), respectively. 30,000 leukocytes per sample were acquired in order to achieve a high precision. We hypothesized a normal distribution of the analyzed frequencies evaluating data from mutants and controls by a two-tailed Student’s t-test, and a significance level of 5%.

### Energy Metabolism

Energy expenditure was monitored by indirect calorimetry (SM-MARS8x, Sable Systems, Las Vegas, USA). High precision CO_2_ and O_2_ sensors measured the difference in CO_2_ and O_2_ concentrations in air volumes flowing through reference and animal cages. The amount of oxygen consumed was calculated by recording airflow through the cages and measuring gas concentrations in parallel. Data for oxygen consumption are expressed as ml O_2_/h/animal. The system also monitored CO_2_ production; therefore, the respiratory exchange ratio (RER) and heat production could be calculated (ratio VCO_2_/VO_2_). Heat production (HP) was calculated from VO_2_ and RER using the formula: HP [mW] = (4.44+1.43×RER)×VO_2_ [ml/h]. The test was performed at 23°C with a 12∶12 hours light/dark cycle in the room (lights on 06∶30 CET, lights off 18∶30 CET). Paper tissue was provided as bedding material. Each mouse was placed individually in the respirometric cages for a period of 21 hours (from 14∶00 CET to 11∶00 CET next day) with free access to food and water. Respirometric cages were set up in a ventilated cabinet continuously supplied with an overflow of fresh air from outside. In addition to gas exchange, body mass before and after the trial was measured. Before returning the mice to their home cage, rectal body temperature was also determined. Food intake was monitored by weighing and re-weighing the feeder before and after the indirect calorimetry test period. 5–10 mice per group (genotype/sex) were analyzed.

Two-way ANOVA (SigmaStat, Jandel Scientific) was used to test for statistically significant differences between strains and sexes. For oxygen consumption, a Linear Regression Model was applied with sex and genotype as main factors and body mass as an additional variable to account for the confounding effect of body mass on energy metabolism parameters.

### Pathology

Mice received in the laboratory of pathology at the GMC were sacrificed with CO_2_. The animals were analyzed macroscopically and weighed (http://eulep.pdn.cam.ac.uk/Necropsy_of_the_Mouse/index.php). The body and heart weights were determined. The tibia, thymus, spleen, kidney and left lobe of the liver were measured. The tibia length was determined from the left tibia of the mouse using a ruler. Heart weight was normalized by dividing the heart weight value (grams) either by body weight (grams) or tibia length (millimeters) and multiplying by 1000. A complete pathological analysis was performed on 6–9 mice per group, and 2–6 additional mice per group were analyzed in follow-up studies.

Following a complete dissection, all organs were fixed in 4% buffered formalin and embedded in paraffin for histological examination. Four-µm-thick sections from skin, heart, muscle, lung, brain, cerebellum, thymus, spleen, cervical lymph nodes, thyroid, parathyroid, adrenal gland, stomach, intestine, liver, pancreas, kidney, reproductive organs, and urinary bladder were cut and stained with hematoxylin and eosin (H&E). In addition, for brain sections, immunohistochemistry (IHC) was performed with an automated immunostainer (Ventana Medical Systems, Inc., Tucson AZ). The slides were deparaffinized and rehydrated. Heat-induced antigen retrieval was performed and primary antibodies against glial fibrillary acidic protein (GFAP, DakoCytomation, Hamburg, Germany; Z0334) and S-100 protein (DakoCytomation, Hamburg, Germany; Z0311) were used as glial cell markers. Staining for the JNCL-hallmark storage material was performed using anti-subunit c antibody (8357), as previously described [8], [93].

Brush cytological preparations of bone marrow from sacrificed mice were performed as described [94]. Tibias for subsequent bone histology were harvested from freshly sacrificed mice, formalin fixed for 48 hours, decalcified in 0.5 M EDTA, pH = 8.0, for 7 days, and paraffin embedded.

Staining for ferric iron was performed using the Accustain Iron Stain kit (Sigma) on snap-frozen, methanol-fixed organ sections and air-dried, methanol-fixed bone marrow cytology specimens.

### Immunofluorescence and Electron Microscopy

For immunofluorescence detection of epithelial cell markers, epididymides (from at least 3 mice/genotype) were fixed in paraformaldehyde, lysine, and periodate (PLP) fixative, as described previously [95], [96], [97]. Cryostat sections were double-labeled using an affinity-purified chicken antibody against the B1 subunit of the V-ATPase (a marker of narrow and clear cells) and an affinity-purified rabbit antibody against aquaporin 9 (AQP9) (a marker of principal cells), as previously described [95], [96], [97].

For electron microscopy (EM), small pieces of epididymis tissues were fixed in 2.0% glutaraldehyde in 0.1 M sodium cacodylate buffer, pH 7.4 (Electron Microscopy Sciences, Hatfield, PA) overnight at 4 C. Samples from at least 2 mice per genotype were analyzed by EM. They were rinsed in buffer, post-fixed in 1.0% osmium tetroxide in cacodylate buffer for one hour at room temperature, rinsed in buffer again and dehydrated through a graded series of ethanol to 100%. They were then infiltrated with Epon resin (Ted Pella, Redding, CA) in a 1∶1 solution of Epon:ethanol. The following day they were placed in fresh Epon for several hours and then embedded in Epon overnight at 60 C. Thin sections were cut on a Reichert Ultracut E ultramicrotome, collected onto formvar-coated grids, stained with uranyl acetate and lead citrate and examined in a JEOL JEM 1011 transmission electron microscope at 80 kV. Images were collected using an AMT digital imaging system (Advanced Microscopy Techniques, Danvers, MA).

### Clinical Chemistry and Hematology

For the determination of blood-based parameters, blood samples were collected from the retro-bulbar sinus of isoflurane-anesthetized mice into Li-heparin-coated or EDTA-coated tubes (KABE; Nümbrecht, Germany). EDTA-blood samples were placed on a rotary agitator and used for hematological analyses, while blood samples collected in Li-heparin-coated tubes were stored at room temperature for one to two hours before being separated into cells and plasma by centrifugation (10 Min, 5000×g, at 8°C).

The plasma samples for the clinical chemical analyses were diluted 1∶2 with deionized water and analyzed using an Olympus AU 400 autoanalyzer and adapted reagents from Beckman-Coulter (Krefeld, Germany). To determine the peripheral blood cell count, EDTA-blood samples were used to measure basic hematological parameters with an ABC-Animal Blood Counter (Scil Animal Care Company GmbH; Viernheim, Germany) using the settings defined for laboratory mouse blood.

Alternatively, 100 ul of peripheral blood was collected from the facial artery of unanesthetized, manually restrained mice, into EDTA-coated Vacutainer tubes (StatSampler, Fisher Scientific) and used to perform automated complete blood counts within the MGH-Center for Comparative Medicine Clinical Pathology Laboratory (on a HemaTrue Veterinary Hematology Analyzer, Heska Corporation, Loveland, CO) and for peripheral blood smears. Reticulocytes were visualized by new methylene blue staining, and absolute reticulocyte counts were determined by manual counts by investigators who were blinded to genotype. Wright-Giemsa staining of peripheral blood smears was performed by standard methods. Vacuolated lymphocytes were quantified from at least 3 representative mice per genotype by manually counting from Wright-Giemsa stained blood smears under a light microscope by an investigator blinded to genotype. Values represent mean ± standard deviation.

Two separate samples from 9–10 mice per group (genotype/sex) were analyzed in the primary screen, and samples from an additional 3–8 mice per group (genotype/sex) were analyzed in follow-up studies.

Clinical chemistry and hematology data were tested for influences of genotype, sex and their interaction using a two-way ANOVA (SigmaStat 3.1). Post-hoc tests were performed either using the Welsh-T-test, with multiple test correction according to the Holm-Sidac method in SigmaStat 3.1, or using the Welsh-Test in Excel, without multiple testing correction.

## Supporting Information

Figure S1 **Open field behavior of** ***Cln3^Δex7/8^*** **mice**. Littermate control (*Cln3^+/+^*, n = 9 males, 10 females), heterozygous (*Cln3^+/^* ^***Δ****ex7/8*^, n = 9 males, 10 females) and homozygous (*Cln3* ***^Δ^*** ^*ex7/8/****Δ****ex7/8*^, n = 10 males, 10 females) mice were tested in an open field arena for 20 minutes, and distance traveled (centimeters = cm), rearing frequency (#) and time spent in the centre were recorded (expressed as % of total time). The bar graphs depict the mean values ± SEM for *Cln3^+/+^* (solid black bars), *Cln3^+/^* ^***Δ****ex7/8*^ (black and white striped bars) and *Cln3* ***^Δ^*** ^*ex7/8/****Δ****ex7/8*^ (solid white bars) mice. Habituation is also shown, which was examined by plotting mean total distance travelled (±SEM) at 5-minute intervals for a total of 20 minutes. Males and females were analyzed and are shown separately. No dramatic genotypic differences in behavior in the open field analyses were observed. However, we noted that the heterozygotes tended to spend more time in the centre of the arena, and there was a trend of reduced habituation over the 20-minute trial for both homozygous *Cln3* ***^Δ^*** ^*ex7/8*^ male and female mice (circles in bottom graphs), compared to wild-type (squares in bottom graphs) or heterozygous *Cln3* ***^Δ^*** ^*ex7/8*^ littermates (triangles in bottom graphs) (ANOVA, p = 0.059 for females and p = 0.096 for males).
(TIF)

Figure S2 **Grip strength measurement of** ***Cln3^Δex7/8^*** **mice.** Strength of the mice with either 2 paws (left graph) or 4 paws (right graph) grasping a horizontal metal grid was measured. Shown are the means (± SEM) for each genotype group. Values were calculated from the means of the individual mice, each tested in triplicate. Males and females were analyzed separately due to sex differences in grip strength. 9–10 mice per group (genotype/sex) were analyzed.
(TIF)

Figure S3 **Rotarod performance of** ***Cln3^Δex7/8^*** **mice.** Bar graphs depict the mean ± SEM of the latency to fall (seconds = sec) from the accelerating rotarod apparatus. Mice were tested on three consecutive days. Motor learning was equally evident over the three days for each of the genotypes: *Cln3^+/+^* data points are represented by circles, *Cln3^+/^* ^***Δ****ex7/8*^ data points are represented by squares, and *Cln^Δx7/8/Δex7/8^* data points are represented by triangles. Latencies to fall significantly increased for all genotypes over the 3 days (p<0.001), in a manner that did not differ by genotype. Data from males and females are shown separately because significant sex differences across the 3-day trial period were observed (ANOVA, interaction of day and sex, p<0.05). No genotypic differences were observed in performance on the accelerating rotarod. 9–10 mice per group (genotype/sex) were analyzed.
(TIF)

Figure S4 **Normalized brain weights from 20-week old** ***Cln3^Δex7/8^*** **mice.** Brain weights (mg) from wild-type (*Cln3^+/+^*) and homozygous (*Cln3^Δex7/8/^* ^***Δ****ex7/8*^) littermate mice, normalized to body weights (g), are shown. Values for males and females are shown separately. The horizontal bar represents the mean and the error bars represent SEM. Circles represent *Cln3^+/+^* values and triangles represent *Cln3* ***^Δ^*** ^*ex7/8/Δex7/8*^ values. Brain weight values shown were pooled from mice inbred on the C57Bl6/NCrl and the C57Bl6/J backgrounds, which do not significantly differ. 5–8 mice per group (genotype/sex) were analyzed; circles and triangles represent datapoints from individual mice.
(TIF)

Figure S5 **Brain morphology and storage material assessment of 20-week old** ***Cln3^Δex7/8^*** **mice.** Representative micrographs of H&E stained and subunit c-immunostained serial brain sections from wild-type (*Cln3^+/+^*) and homozygous mutant mice (*Cln3^Δex7/8/Δex7/8^*) are shown. CA3, hippocampal pyradmidal cell layer CA3, DG = dentate gyrus, H = medial habenular nucleus, AD = anterodorsal thalamic nucleus, VL = ventrolateral thalamic nucleus, Str = striatum, Pir = pyriform cortex, BLA = basolateral amygdaloid nucleus, BMA = basomedial amygdaloid nucleus. Scale bars = 200 µm. Boxed regions are shown digitally zoomed in right column.
(TIF)

Figure S6 **S100 immunostaining of 20-week old** ***Cln3^Δex7/8^*** **mouse brain.** Representative images of S100 immunostained wild-type (*Cln3^+/+^*) and homozygous (*Cln3^Δex7/8/Δex7/8^*) littermate mouse brain sections (20-weeks of age) are shown. The CA1-CA3 regions of the pyramidal cell layer and the dentate gyrus of the hippocampus (top panels, scale bar = 200 µm) and the thalamus (bottom panels, scale bar = 200 µm) are shown. The CA1 region is also shown at higher magnification (scale bar = 50 µm). Note the overall darker stain, particularly in the neuropil, in the homozygous *Cln3* ***^Δ^*** ^*ex7/8*^ images. The overall number of S100-positive astrocytes does not appear to differ between 20-week old wild-type and homozygous *Cln3* ***^Δ^*** ^*ex7/8*^ mice.
(TIF)

Figure S7 **Blood pressure and pulse rate of** ***Cln3^Δex7/8^*** **mice.** Mean ± SEM blood pressure and pulse rate, measured using a tail-cuff system, are shown for wild-type (*Cln3^+/+^*), heterozygous (*Cln3^+/^* ^***Δ****ex7/8*^), and homozygous (*Cln3* ***^Δ^*** ^*ex7/8/****Δ****ex7/8*^) littermate mice (n = 6−8 mice per group). No genotypic differences were observed.
(TIF)

Figure S8 **Serum Nt-proANP levels in** ***Cln3^Δex7/8^*** **mice.** Serum Nt-proANP levels for male and female wild-type (*Cln3^+/+^*), heterozygous (*Cln3^+/^* ^***Δ****ex7/8*^), and homozygous (*Cln3* ***^Δ^*** ^*ex7/8/Δex7/8*^) littermate mice are shown (n = 9−10 mice per group). Horizontal bars represent the mean and error bars represent SEM. No significant differences were observed. nmol/l = nanomoles per liter
(TIF)

Figure S9 **Spleen and liver analysis in** ***Cln3^Δex7/8^*** **mice.** The ratios of spleen weight (A) and liver weight (B) to total body weight (grams, ‘g’) in 12-week-old mice of each genotype are shown. No significant pairwise differences were detected by an unpaired, two-way *t* test. Circles, squares and triangles represent datapoints from individual mice. (C) Representative H&E-stained sections of spleen from wild-type and homozygous mutant mice (scale bar = 400 µm; insets represent digitally zoomed boxed regions). (D) Representative H&E-stained sections of liver from wild-type (*Cln3^+/+^*), and homozygous (*Cln3^Δex7/8/Δex7/8^*) mutant littermate mice are shown (scale bar = 100 µm).
(TIF)

Table S1 **Laser interference biometry, funduscopy, and slit lamp microscopy data from the eye screen of** ***Cln3^Δex7/8^*** **mice.** For each parameter, the mean values ± SEM or the frequency of the total mice displaying the described features are indicated for each genotype group. Male and female data are shown separately. ‘n’ for each parameter was between 6 and 10 mice, as specifically indicated in the table. No significant genotypic differences were observed in the eye screen parameters.
(DOC)

Table S2 **Metabolic parameters recorded in the primary screen of** ***Cln3^Δex7/8^*** **mice.** Results for males (13 weeks of age) and females (14 weeks of age) are shown separately. No genotypic differences were observed in body weight or in the food consumption, respiratory exchange ratio (RER), and activity parameters in a 12 hour light:12 hour dark cycle indirect calorimetry assay (ANOVA, p>0.05). However, rectal body temperature, measured at the end of the light/dark cycle, was elevated in both heterozygous and homozygous *Cln3* ***^Δ^*** ^*ex7/8*^ mice, compared to wild-type littermates (ANOVA, p<0.001). Minimum oxygen (O_2_) consumption was also elevated in heterozygous and homozygous *Cln3* ***^Δ^*** ^*ex7/8*^ mice, compared to wild-type littermates (ANOVA, p<0.05). Mean oxygen consumption tended to be higher as well, but this was not significant by ANOVA. Bolded rows highlight parameters that differed by genotype. 5–10 mice per group (genotype/sex) were analyzed, as indicated.
(DOC)

Table S3 **Echocardiography analysis of** ***Cln3^Δex7/8^*** **mice.** Cardiovascular function parameters of wild-type (*Cln3^+/+^*), heterozygous (*Cln3^+/Δex7/8^*), and homozygous (*Cln3^Δex7/8/Δex7/8^*) littermate mice, measured by echocardiography, are shown. LVID = left ventricular internal dimension, mm = millimeters, bpm = beats per minute, ml = milliliters, % = percent. Values represent the mean ± SEM. No genotypic differences in echocardiography parameters were observed. 6–8 mice per group (genotype/sex) were analyzed, as indicated.
(DOC)

Table S4 **Blood analytes and hematological parameters in** ***Cln3*** *^Δ^* ^***ex7/8***^ **mice.** The results of clinical chemistry and hematological analysis of blood isolated from 12- to 19-week-old wild-type (*Cln3^+/+^*), heterozygous (*Cln3^+/^* ^***Δ****ex7/8*^), and homozygous (*Cln3^Δex7/8/Δex7/8^*) littermate mice are shown, with significantly different values indicated in bold. Each row of values represents an independent set of measurements. Data represent mean ± SEM. *p<0.05, **p<0.01 (2-way ANOVA for each set of measurements). For the statistical analysis of ferritin levels, one mouse from the group of heterozygous (*Cln3^+/Δex7/8^*) females was excluded as an outlier. Non-italicized values were determined at the German Mouse Clinic (GMC). Italicized values were determined at Massachusetts General Hospital and were derived from peripheral blood isolated from a separate cohort of mice from those analyzed in the primary screen at the GMC. The ∼5–6 fL offset in MCV measurements taken at the two different sites, as well as the ∼6–7% offset in RDW measurements, are likely due to differences in the automated analyzers used. Inorg. = inorganic, NEFA = non-esterified fatty acids, LDH = lactate dehydrogenase, ALAT = alanine transaminase, ASAT = aspartate transaminase, ALP = alkaline phosphatase, WBC = white blood cell count, RBC = red blood cell count, PLT = platelet count, MCV = mean corpuscular volume, MCH = mean corpuscular hemoglobin, MCHC = mean cell hemoglobin concentration, RDW = red cell distribution width, MPV = mean platelet volume, Retic. = reticulocyte. Two separate samples from 9–10 mice per group (genotype/sex) were analyzed in the primary screen, and samples from an additional 3–8 mice per group (genotype/sex) were analyzed in follow-up screens.
(XLS)
