# Supplementary figures and images for: Large-Scale Phenotyping of an Accurate Genetic Mouse Model of JNCL Identifies Novel Early Pathology Outside the Central Nervous System
Source: PLoS One. 2012 Jun 6;7(6):e38310. doi: 10.1371/journal.pone.0038310 (PMC3368842; doi:10.1371/journal.pone.0038310)

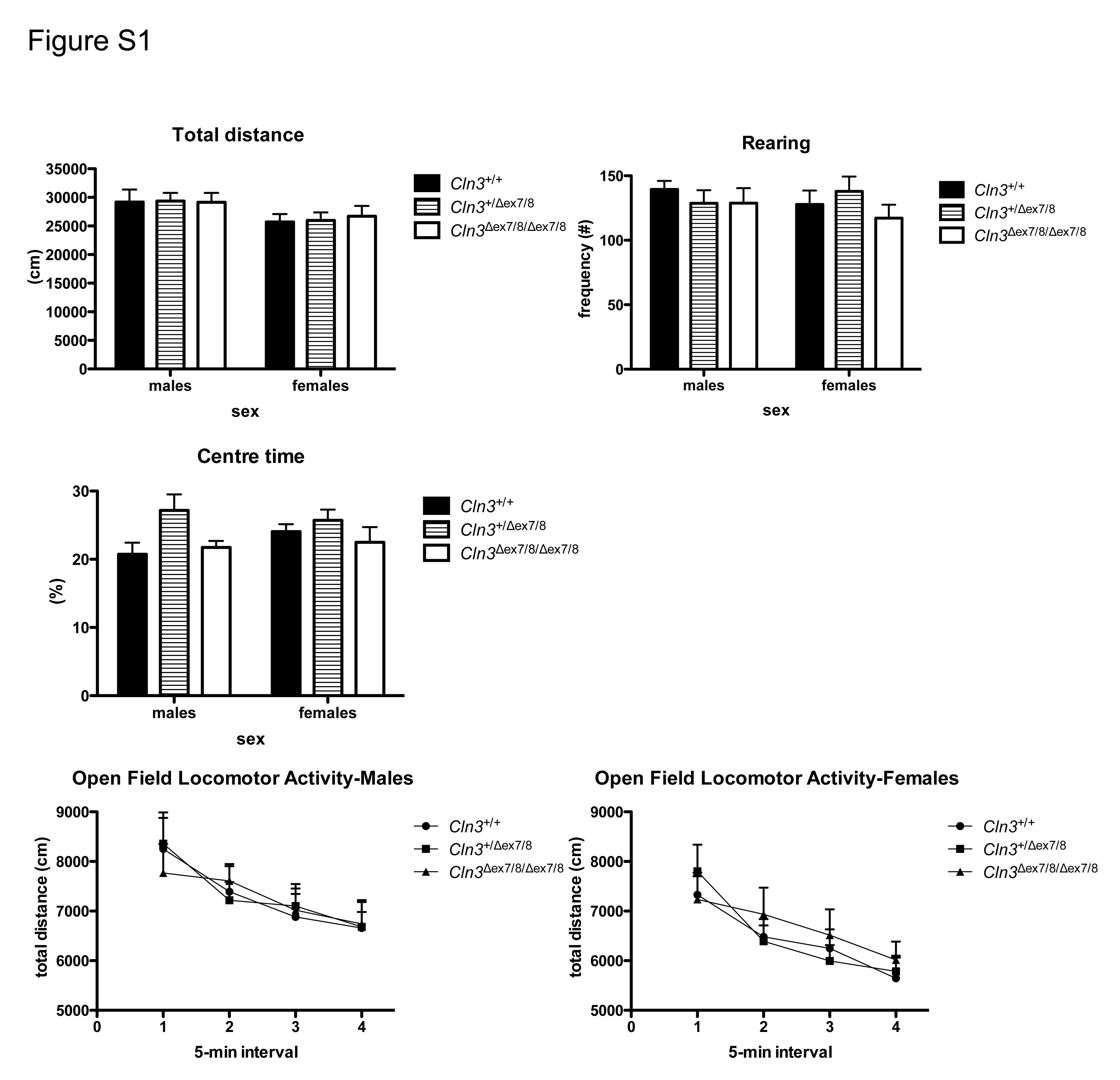

Supplement: Figure S1 — Open field behavior of Cln3Δex7/8 mice. Littermate control (Cln3+/+, n = 9 males, 10 females), heterozygous (Cln3+/ Δex7/8, n = 9 males, 10 females) and homozygous (Cln3 Δ ex7/8/Δex7/8, n = 10 males, 10 females) mice were tested in an open field arena for 20 minutes, and distance traveled (centimeters = cm), rearing frequency (#) and time spent in the centre were recorded (expressed as % of total time). The bar graphs depict the mean values ± SEM for Cln3+/+ (solid black bars), Cln3+/ Δex7/8 (black and white striped bars) and Cln3 Δ ex7/8/Δex7/8 (solid white bars) mice. Habituation is also shown, which was examined by plotting mean total distance travelled (±SEM) at 5-minute intervals for a total of 20 minutes. Males and females were analyzed and are shown separately. No dramatic genotypic differences in behavior in the open field analyses were observed. However, we noted that the heterozygotes tended to spend more time in the centre of the arena, and there was a trend of reduced habituation over the 20-minute trial for both homozygous Cln3 Δ ex7/8 male and female mice (circles in bottom graphs), compared to wild-type (squares in bottom graphs) or heterozygous Cln3 Δ ex7/8 littermates (triangles in bottom graphs) (ANOVA, p = 0.059 for females and p = 0.096 for males). (TIF) [file pone.0038310.s001.tif]

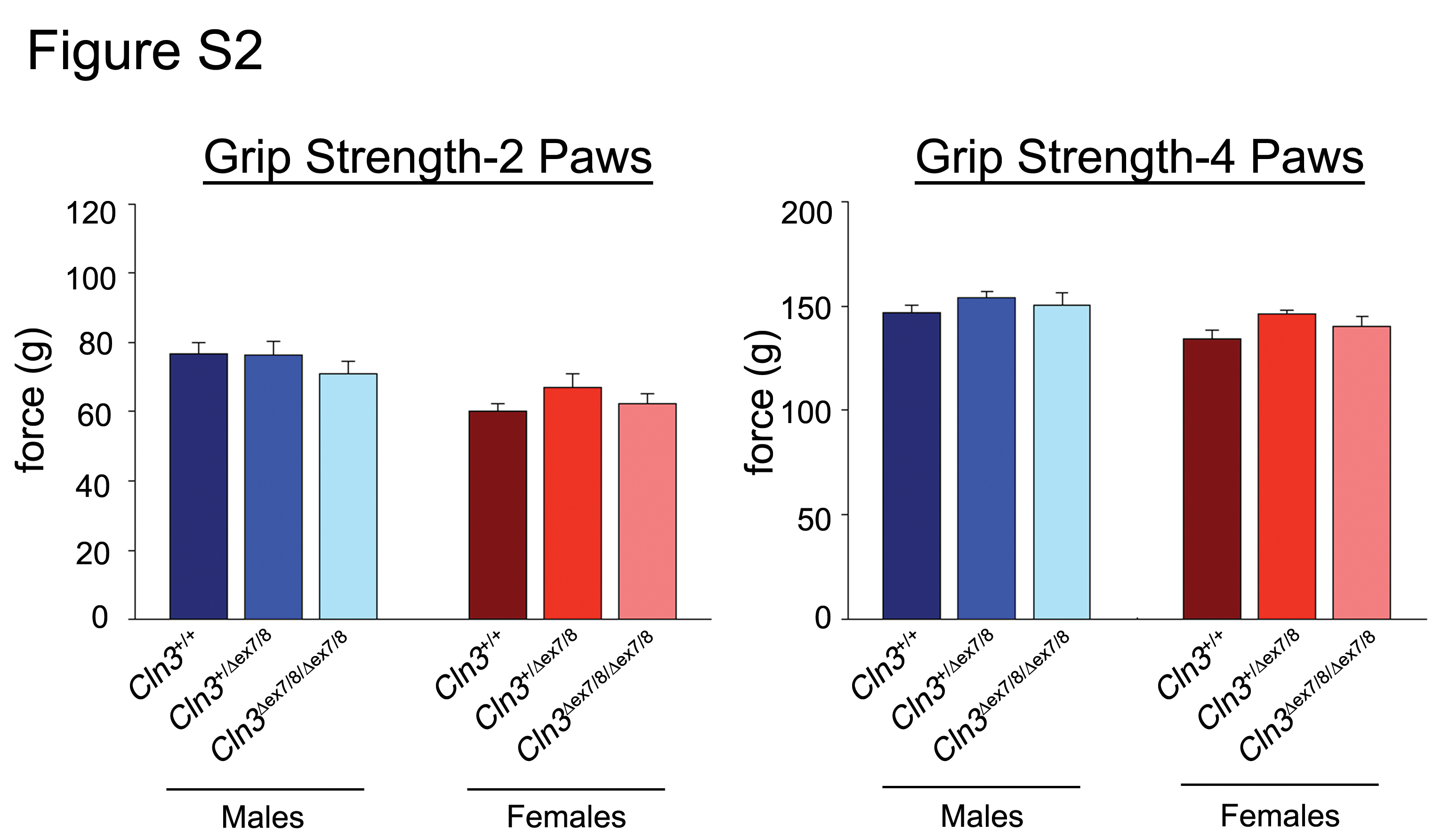

Supplement: Figure S2 — Grip strength measurement of Cln3Δex7/8 mice. Strength of the mice with either 2 paws (left graph) or 4 paws (right graph) grasping a horizontal metal grid was measured. Shown are the means (± SEM) for each genotype group. Values were calculated from the means of the individual mice, each tested in triplicate. Males and females were analyzed separately due to sex differences in grip strength. 9–10 mice per group (genotype/sex) were analyzed. (TIF) [file pone.0038310.s002.tif]

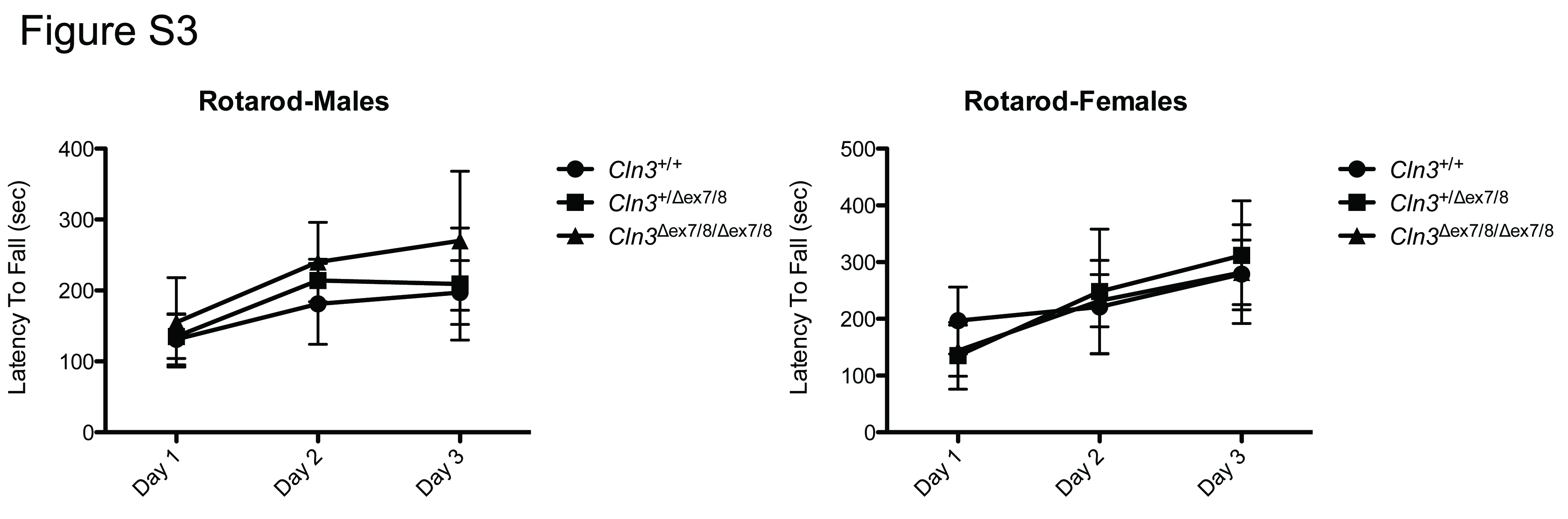

Supplement: Figure S3 — Rotarod performance of Cln3Δex7/8 mice. Bar graphs depict the mean ± SEM of the latency to fall (seconds = sec) from the accelerating rotarod apparatus. Mice were tested on three consecutive days. Motor learning was equally evident over the three days for each of the genotypes: Cln3+/+ data points are represented by circles, Cln3+/ Δex7/8 data points are represented by squares, and ClnΔx7/8/Δex7/8 data points are represented by triangles. Latencies to fall significantly increased for all genotypes over the 3 days (p<0.001), in a manner that did not differ by genotype. Data from males and females are shown separately because significant sex differences across the 3-day trial period were observed (ANOVA, interaction of day and sex, p<0.05). No genotypic differences were observed in performance on the accelerating rotarod. 9–10 mice per group (genotype/sex) were analyzed. (TIF) [file pone.0038310.s003.tif]

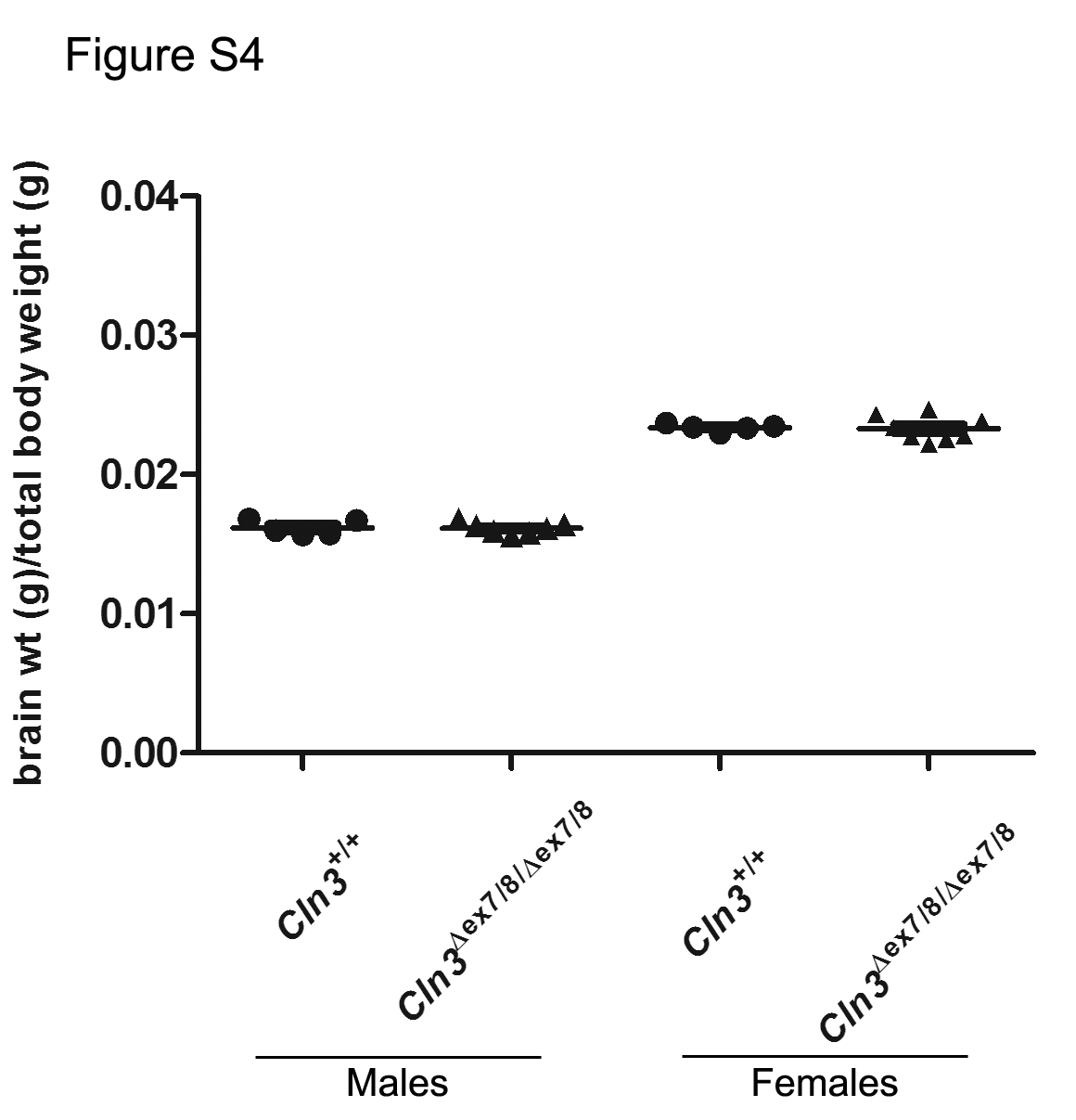

Supplement: Figure S4 — Normalized brain weights from 20-week old Cln3Δex7/8 mice. Brain weights (mg) from wild-type (Cln3+/+) and homozygous (Cln3Δex7/8/ Δex7/8) littermate mice, normalized to body weights (g), are shown. Values for males and females are shown separately. The horizontal bar represents the mean and the error bars represent SEM. Circles represent Cln3+/+ values and triangles represent Cln3 Δ ex7/8/Δex7/8 values. Brain weight values shown were pooled from mice inbred on the C57Bl6/NCrl and the C57Bl6/J backgrounds, which do not significantly differ. 5–8 mice per group (genotype/sex) were analyzed; circles and triangles represent datapoints from individual mice. (TIF) [file pone.0038310.s004.tif]

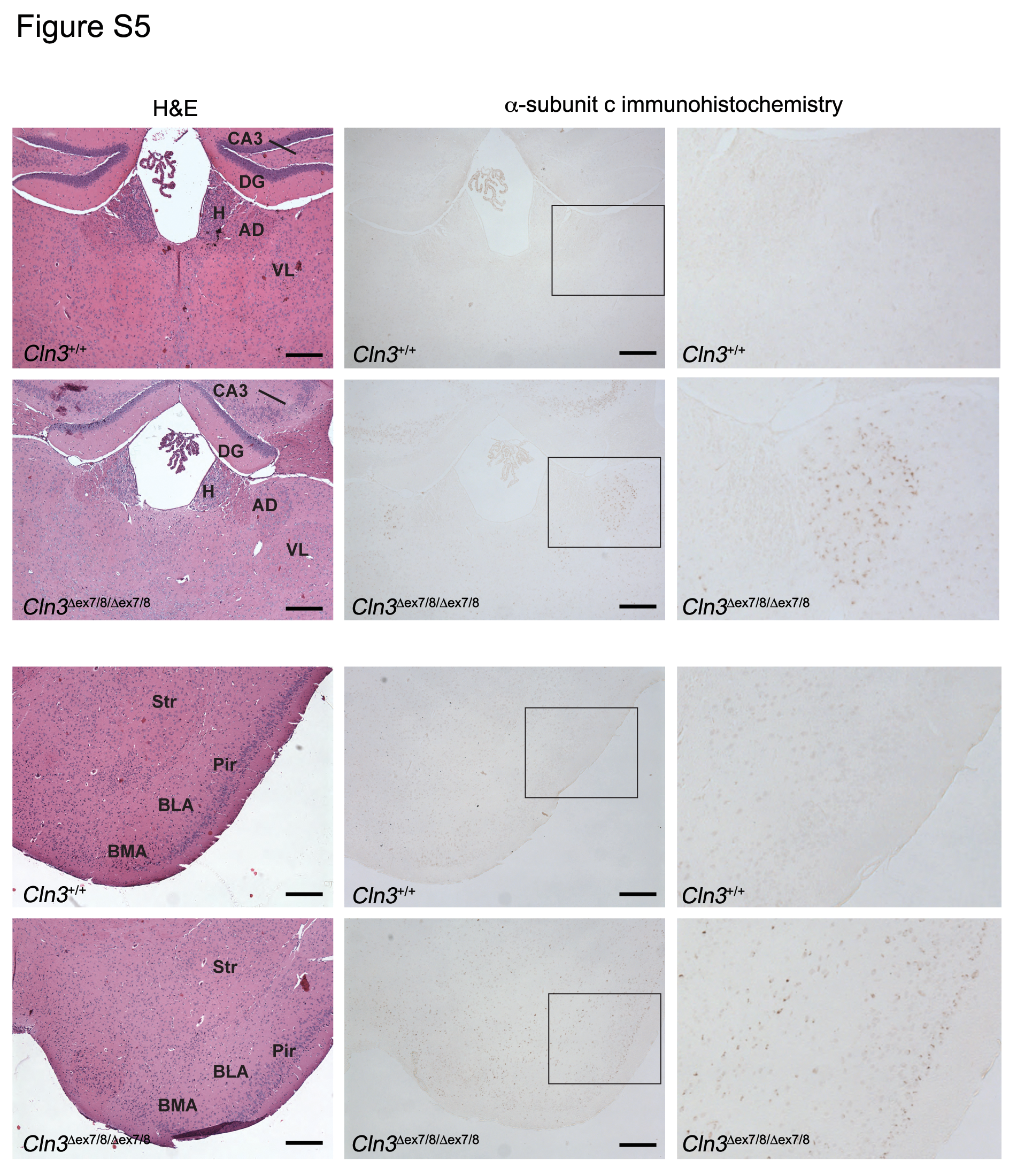

Supplement: Figure S5 — Brain morphology and storage material assessment of 20-week old Cln3Δex7/8 mice. Representative micrographs of H&E stained and subunit c-immunostained serial brain sections from wild-type (Cln3+/+) and homozygous mutant mice (Cln3Δex7/8/Δex7/8) are shown. CA3, hippocampal pyradmidal cell layer CA3, DG = dentate gyrus, H = medial habenular nucleus, AD = anterodorsal thalamic nucleus, VL = ventrolateral thalamic nucleus, Str = striatum, Pir = pyriform cortex, BLA = basolateral amygdaloid nucleus, BMA = basomedial amygdaloid nucleus. Scale bars = 200 µm. Boxed regions are shown digitally zoomed in right column. (TIF) [file pone.0038310.s005.tif]

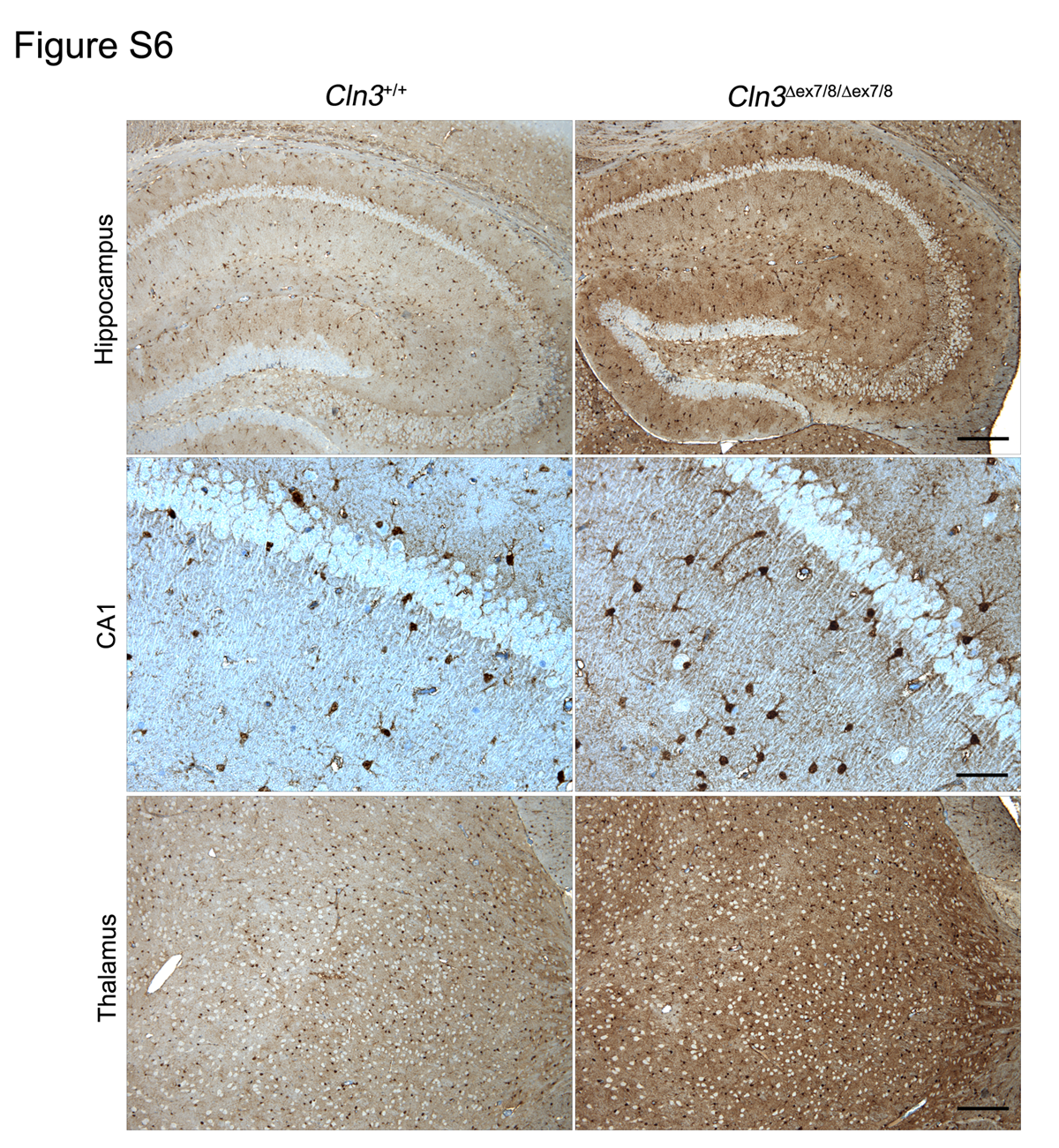

Supplement: Figure S6 — S100 immunostaining of 20-week old Cln3Δex7/8 mouse brain. Representative images of S100 immunostained wild-type (Cln3+/+) and homozygous (Cln3Δex7/8/Δex7/8) littermate mouse brain sections (20-weeks of age) are shown. The CA1-CA3 regions of the pyramidal cell layer and the dentate gyrus of the hippocampus (top panels, scale bar = 200 µm) and the thalamus (bottom panels, scale bar = 200 µm) are shown. The CA1 region is also shown at higher magnification (scale bar = 50 µm). Note the overall darker stain, particularly in the neuropil, in the homozygous Cln3 Δ ex7/8 images. The overall number of S100-positive astrocytes does not appear to differ between 20-week old wild-type and homozygous Cln3 Δ ex7/8 mice. (TIF) [file pone.0038310.s006.tif]

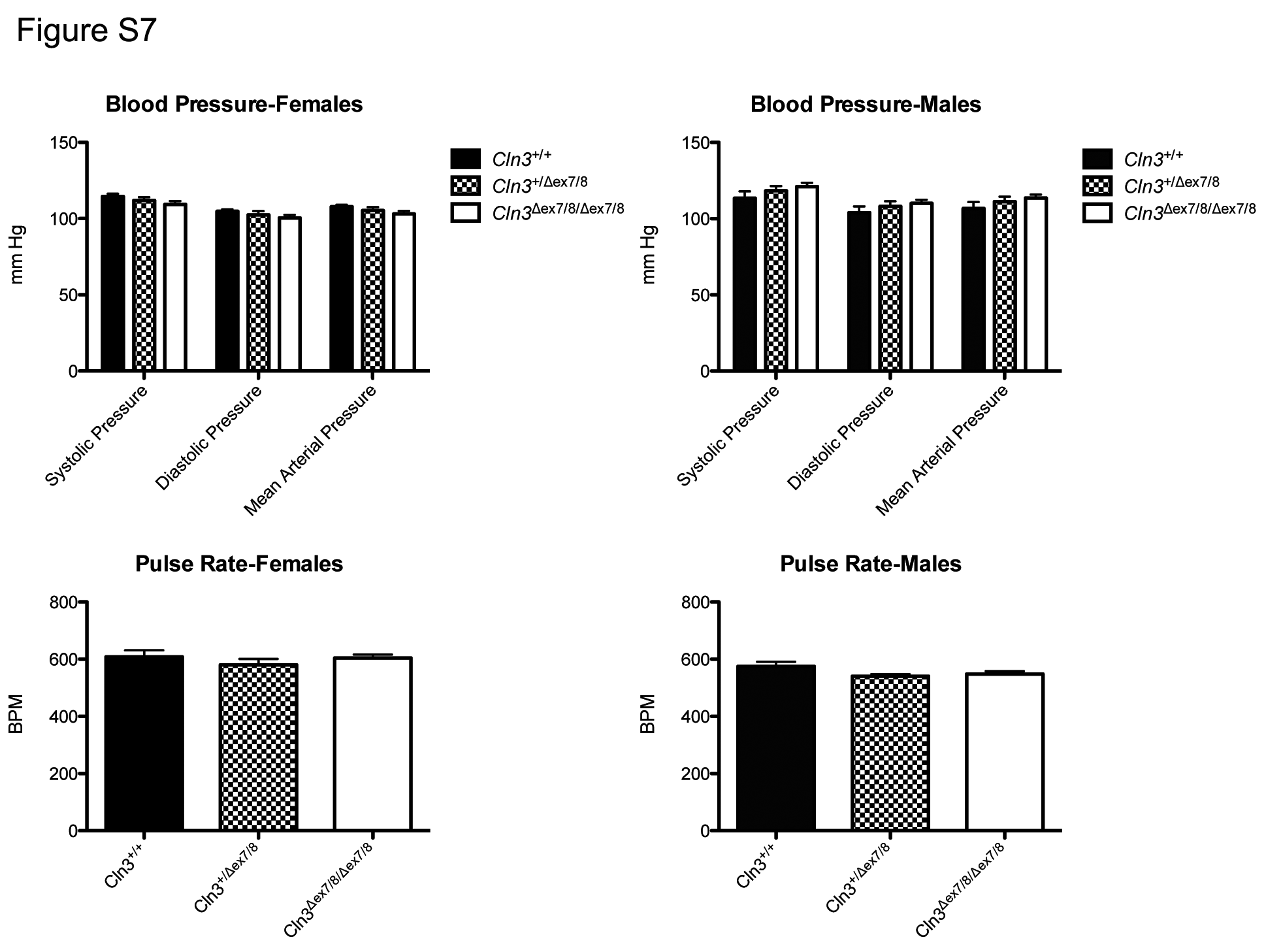

Supplement: Figure S7 — Blood pressure and pulse rate of Cln3Δex7/8 mice. Mean ± SEM blood pressure and pulse rate, measured using a tail-cuff system, are shown for wild-type (Cln3+/+), heterozygous (Cln3+/ Δex7/8), and homozygous (Cln3 Δ ex7/8/Δex7/8) littermate mice (n = 6−8 mice per group). No genotypic differences were observed. (TIF) [file pone.0038310.s007.tif]

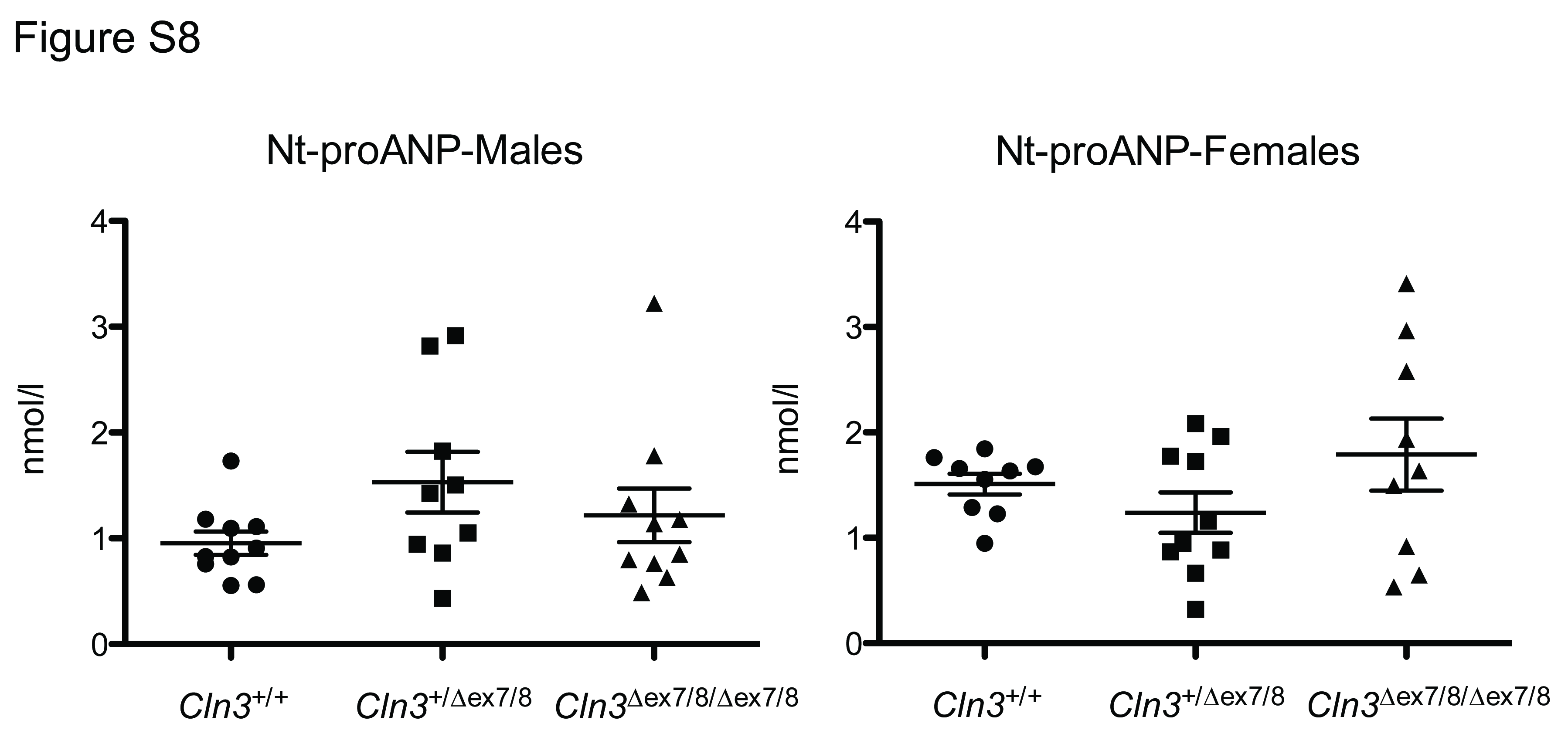

Supplement: Figure S8 — Serum Nt-proANP levels in Cln3Δex7/8 mice. Serum Nt-proANP levels for male and female wild-type (Cln3+/+), heterozygous (Cln3+/ Δex7/8), and homozygous (Cln3 Δ ex7/8/Δex7/8) littermate mice are shown (n = 9−10 mice per group). Horizontal bars represent the mean and error bars represent SEM. No significant differences were observed. nmol/l = nanomoles per liter (TIF) [file pone.0038310.s008.tif]

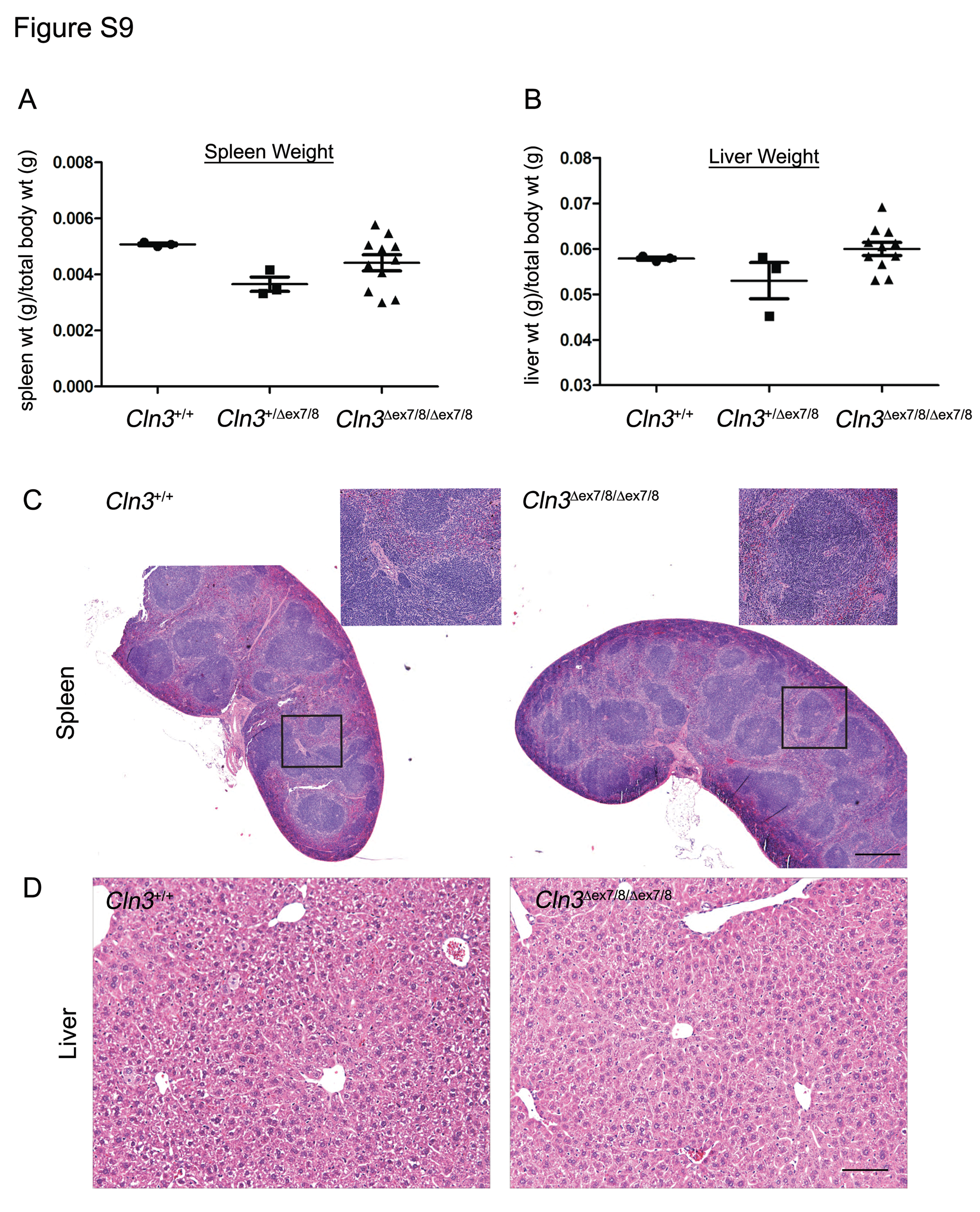

Supplement: Figure S9 — Spleen and liver analysis in Cln3Δex7/8 mice. The ratios of spleen weight (A) and liver weight (B) to total body weight (grams, ‘g’) in 12-week-old mice of each genotype are shown. No significant pairwise differences were detected by an unpaired, two-way t test. Circles, squares and triangles represent datapoints from individual mice. (C) Representative H&E-stained sections of spleen from wild-type and homozygous mutant mice (scale bar = 400 µm; insets represent digitally zoomed boxed regions). (D) Representative H&E-stained sections of liver from wild-type (Cln3+/+), and homozygous (Cln3Δex7/8/Δex7/8) mutant littermate mice are shown (scale bar = 100 µm). (TIF) [file pone.0038310.s009.tif]
