# Supplementary material for: Large-Scale Phenotyping of an Accurate Genetic Mouse Model of JNCL Identifies Novel Early Pathology Outside the Central Nervous System
Source: PLoS One. 2012 Jun 6;7(6):e38310. doi: 10.1371/journal.pone.0038310 (PMC3368842; doi:10.1371/journal.pone.0038310)
Supplement: Table S1 — Laser interference biometry, funduscopy, and slit lamp microscopy data from the eye screen of Cln3Δex7/8 mice. For each parameter, the mean values ± SEM or the frequency of the total mice displaying the described features are indicated for each genotype group. Male and female data are shown separately. ‘n’ for each parameter was between 6 and 10 mice, as specifically indicated in the table. No significant genotypic differences were observed in the eye screen parameters. (DOC) [file pone.0038310.s010.doc]

**Table S1.** **Laser interference biometry, funduscopy, and slit lamp microscopy data from the eye screen of *Cln3*^∆ex7/8^ mice.**

| **Parameter** | **Males** | | | **Females** | | |
| --- | --- | --- | --- | --- | --- | --- |
|  | ***Cln3*^+/+^** | ***Cln3*^+/∆ex7/8^** | ***Cln3*^∆ex7/8/∆ex7/8^** | ***Cln3*^+/+^** | ***Cln3*^+/∆ex7/8^** | ***Cln3*^∆ex7/8/∆ex7/8^** |
| Axial eye length (mm) | 3.6± .03 (n=6) | 3.6± .03 (n=7) | 3.6± .04 (n=7) | 3.6± .03 (n=7) | 3.6± .03 (n=7) | 3.6± .04 (n=7) |
| Axial eye length/body length | 0.036± .0005 (n=6) | .037± .0006 (n=7) | .036± .0004 (n=7) | .041± .0008  (n=7) | .04± .0010  (n=7) | .042± .0007  (n=7) |
| Funduscopy-White dots detected | 1/10 | 1/9 | 4/10 | 1/9 | 0/10 | 0/10 |
| Funduscopy-Enlarged optic disc | 0/10 | 0/9 | 0/10 | 1/10 | 0/10 | 0/10 |
| Funduscopy-Vessel alternations | 0/10 | 0/9 | 0/10 | 0/10 | 0/10 | 0/10 |
| Slit Lamp Biomicroscopy-nuclear flecks/nuclear cloudy opacity | 10/10 | 9/9 | 10/10 | 10/10 | 10/10 | 10/10 |
| Slit Lamp Biomicroscopy-posterior pole opacity | 0/10 | 0/9 | 0/10 | 0/10 | 0/10 | 0/10 |
| Slit Lamp Biomicroscopy-microphthalemia | 0/10 | 0/9 | 0/10 | 0/10 | 0/10 | 0/10 |

For each parameter, the mean values ± SEM or the frequency of the total mice displaying the described features are indicated for each genotype group. Male and female data are shown separately. ‘n’ for each parameter was between 6 and 10 mice, as specifically indicated in the table. No significant genotypic differences were observed in the eye screen parameters.
