# Supplementary material for: Large-Scale Phenotyping of an Accurate Genetic Mouse Model of JNCL Identifies Novel Early Pathology Outside the Central Nervous System
Source: PLoS One. 2012 Jun 6;7(6):e38310. doi: 10.1371/journal.pone.0038310 (PMC3368842; doi:10.1371/journal.pone.0038310)
Supplement: Table S2 — Metabolic parameters recorded in the primary screen of Cln3Δex7/8 mice. Results for males (13 weeks of age) and females (14 weeks of age) are shown separately. No genotypic differences were observed in body weight or in the food consumption, respiratory exchange ratio (RER), and activity parameters in a 12 hour light:12 hour dark cycle indirect calorimetry assay (ANOVA, p>0.05). However, rectal body temperature, measured at the end of the light/dark cycle, was elevated in both heterozygous and homozygous Cln3 Δ ex7/8 mice, compared to wild-type littermates (ANOVA, p<0.001). Minimum oxygen (O2) consumption was also elevated in heterozygous and homozygous Cln3 Δ ex7/8 mice, compared to wild-type littermates (ANOVA, p<0.05). Mean oxygen consumption tended to be higher as well, but this was not significant by ANOVA. Bolded rows highlight parameters that differed by genotype. 5–10 mice per group (genotype/sex) were analyzed, as indicated. (DOC) [file pone.0038310.s011.doc]

**Table S2.** **Metabolic parameters recorded in the primary screen of *Cln3*^∆ex7/8^ mice.**

| **Parameter** | **Males (13 weeks old)** | | | **Females (14 weeks old)** | | |
| --- | --- | --- | --- | --- | --- | --- |
|  | ***Cln3*^+/+^** (n=10) | ***Cln3*^+/∆ex7/8^** (n=8) | ***Cln3*^∆ex7/8/∆ex7/8^** (n=10) | ***Cln3*^+/+^** (n=10) | ***Cln3*^+/∆ex7/8^** (n=5) | ***Cln3*^∆ex7/8/∆ex7/8^** (n=5) |
| Body weight  (g) | 26.7±0.5 | 26.4±0.6 | 27±0.6 | 20.9±0.5 | 22.4±0.4 | 21.5±0.3 |
| Rectal body temperature (˚C) | **36.27±0.2** | **36.79±0.1** | **37.04±0.2** | **36.23±0.3** | **36.95±0.3** | **37.13±0.1** |
| Food consumption (g/day) | 6.3±0.9 | 5.0±0.6 | 5.9±0.8 | 5.2±0.3 | 5.4±0.4 | 5.3±0.4 |
| Mean O_2_ consumption (ml/h) | 91.17±1.7 | 93.61±1.8 | 95.83±2.2 | 91.07±3.7 | 92.38±2.9 | 94.4±1.5 |
| RER, mean | 0.86±0.01 | 0.84±0.01 | 0.83±0.01 | 0.83±0.01 | 0.84±0.01 | 0.83±0.01 |
| Minimum O_2_ consumption (ml/h) | **61.04±1.1** | **64.52±1.6** | **63.65±2.1** | **55.75±1.2** | **60.11±2.7** | **63.11±1.2** |
| Activity (counts x 100) | 14.6±5.7 | 9.3±3.3 | 8.4±3.0 | 7.2±2.0 | 11.3±3.4 | 8.4±2.8 |

Results for males (13 weeks of age) and females (14 weeks of age) are shown separately. No genotypic differences were observed in body weight or in the food consumption, respiratory exchange ratio (RER), and activity parameters in a 12 hour light:12 hour dark cycle indirect calorimetry assay (ANOVA, p>0.05). However, rectal body temperature, measured at the end of the light/dark cycle, was elevated in both heterozygous and homozygous *Cln3*^∆ex7/8^ mice, compared to wild-type littermates (ANOVA, p<0.001). Minimum oxygen (O_2_) consumption was also elevated in heterozygous and homozygous *Cln3*^∆ex7/8^ mice, compared to wild-type littermates (ANOVA, p<0.05). Mean oxygen consumption tended to be higher as well, but this was not significant by ANOVA. Bolded rows highlight parameters that differed by genotype. 5-10 mice per group (genotype/sex) were analyzed, as indicated.
