# Supplementary material for: Large-Scale Phenotyping of an Accurate Genetic Mouse Model of JNCL Identifies Novel Early Pathology Outside the Central Nervous System
Source: PLoS One. 2012 Jun 6;7(6):e38310. doi: 10.1371/journal.pone.0038310 (PMC3368842; doi:10.1371/journal.pone.0038310)
Supplement: Table S3 — Echocardiography analysis of Cln3Δex7/8 mice. Cardiovascular function parameters of wild-type (Cln3+/+), heterozygous (Cln3+/Δex7/8), and homozygous (Cln3Δex7/8/Δex7/8) littermate mice, measured by echocardiography, are shown. LVID = left ventricular internal dimension, mm = millimeters, bpm = beats per minute, ml = milliliters, % = percent. Values represent the mean ± SEM. No genotypic differences in echocardiography parameters were observed. 6–8 mice per group (genotype/sex) were analyzed, as indicated. (DOC) [file pone.0038310.s012.doc]

| **Parameter** | **Males** | | | **Females** | | |
| --- | --- | --- | --- | --- | --- | --- |
|  | ***Cln3*^+/+^** (n=8) | ***Cln3*^+/∆ex7/8^** (n=7) | ***Cln3*^∆ex7/8/∆ex7/8^** (n=7) | ***Cln3*^+/+^** (n=6) | ***Cln3*^+/∆ex7/8^** (n=8) | ***Cln3*^∆ex7/8/∆ex7/8^** (n=8) |
| LVID diastolic (mm) | 3.8±0.1 | 3.7±0.1 | 3.8±0.1 | 3.7±0.1 | 3.9±0.1 | 3.6±0.1 |
| LVID systolic (mm) | 2.4±0.2 | 2.3±0.1 | 2.3±0.1 | 2.5±0.2 | 2.7±0.1 | 2.3±0.1 |
| Heart rate (bpm) | 483.4±15.0 | 499.9±8.0 | 474.0±10.5 | 454.1±11.3 | 433.7±13.0 | 449.3±14.9 |
| Fractional shortening (%) | 37.6±2.8 | 38.0±1.9 | 38.6±1.9 | 33.0±2.6 | 31.0±1.4 | 36.5±2.1 |
| LV volume diastolic (ml) | 63.1±3.1 | 59.8±4.8 | 62.0±4.2 | 57.6±3.6 | 64.9±3.6 | 55.2±2.1 |
| LV volume systolic (ml) | 21.1±3.4 | 19.2±2.4 | 19.3±2.4 | 22.6±3.5 | 26.7±2.2 | 18.7±2.3 |
| Ejection fraction (%) | 67.6±3.8 | 68.7±2.3 | 69.3±2.4 | 61.9±3.7 | 59.1±2.1 | 66.8±2.8 |

**Table S3. Echocardiography analysis of *Cln3*^∆ex7/8^ mice.**

Cardiovascular function parameters of wild-type (*Cln3*^+/+^), heterozygous (*Cln3*^+/∆ex7/8^), and homozygous (*Cln3*^∆ex7/8/∆ex7/8^) littermate mice, measured by echocardiography, are shown. LVID=left ventricular internal dimension, mm=millimeters, bpm=beats per minute, ml=milliliters, %=percent. Values represent the mean ± SEM. No genotypic differences in echocardiography parameters were observed. 6-8 mice per group (genotype/sex) were analyzed, as indicated.
